# Deep convolutional architectures for extrapolative forecasts in time-dependent flow problems

**DOI:** 10.1186/s40323-023-00254-y

**Published:** 2023-11-30

**Authors:** Pratyush Bhatt, Yash Kumar, Azzeddine Soulaïmani

**Affiliations:** 1 Department of Mechanical Engineering, Delhi Technological University, P4X9+Q8X, Bawana Rd, Shahbad Daulatpur Village, Rohini, New Delhi, 110042 Delhi India; 2 Department of Mechanical Engineering, École de technologie supérieure, 1100 Rue Notre-Dame W., Montreal, H3C1K3 QC Canada

**Keywords:** Non-intrusive reduced-order modeling, Deep autoencoders, LSTM, TCN, CNN, Time-dependent flow problems

## Abstract

Physical systems whose dynamics are governed by partial differential equations (PDEs) find numerous applications in science and engineering. The process of obtaining the solution from such PDEs may be computationally expensive for large-scale and parameterized problems. In this work, deep learning techniques developed especially for time-series forecasts, such as LSTM and TCN, or for spatial-feature extraction such as CNN, are employed to model the system dynamics for advection-dominated problems. This paper proposes a Convolutional Autoencoder(CAE) model for compression and a CNN future-step predictor for forecasting. These models take as input a sequence of high-fidelity vector solutions for consecutive time steps obtained from the PDEs and forecast the solutions for the subsequent time steps using auto-regression; thereby reducing the computation time and power needed to obtain such high-fidelity solutions. Non-intrusive reduced-order modeling techniques such as deep auto-encoder networks are utilized to compress the high-fidelity snapshots before feeding them as input to the forecasting models in order to reduce the complexity and the required computations in the online and offline stages. The models are tested on numerical benchmarks (1D Burgers’ equation and Stoker’s dam-break problem) to assess the long-term prediction accuracy, even outside the training domain (i.e. extrapolation). The most accurate model is then used to model a hypothetical dam break in a river with complex 2D bathymetry. The proposed CNN future-step predictor revealed much more accurate forecasting than LSTM and TCN in the considered spatiotemporal problems.

## Introduction

Efficient numerical simulations of complex dynamical systems are needed to seek solutions at different times or parameter instances, especially in fluid dynamics. These systems are typically described by a set of parameterized nonlinear partial differential equations (PDEs). Obtaining numerical solutions using a high-fidelity (finite element, finite volume, or finite difference type) computational solver may be extremely expensive, as they must create high-dimensional renderings of the solution to precisely resolve the spatial-temporal multifolds and inherent non-linearities. This method thus becomes inefficient for applications such as optimization and uncertainty quantification, where numerous simulations are required for such analysis. Reduced-order models (ROMs) are suitable substitutions for computationally expensive numerical solvers, as these methods generate a low-ranked structure of the high-dimensional snapshots, which are then utilized to model the spatiotemporal dynamics of the PDE system. Among the various ROM techniques that have been developed, projection-based ROMs are the type employed most extensively. The method involves the generation of a reduced set of basis functions or modes such that their linear superposition effectively overlaps a low-rank approximation of the solutions. Proper Orthogonal Decomposition (POD) is the most popular method among the reduced basis class. POD utilizes singular value decomposition (SVD) to generate an empirical basis of dominant orthonormal modes to obtain an optimum linear subspace in which to project the system-governing PDEs [1, 2]. Availability of the governing equations is necessary to employ intrusive ROM techniques such as the Galerkin projection [3], or the Petrov-Galerkin projection [4], which produce an interpretable ROM defined by high-energy or dominant modes. However, scenarios where the governing equations are unavailable, require the application of data-driven methods, such as non-intrusive ROM (NIROM) [5, 6]. In a NIROM, the expansion coefficients for the reduced solution are obtained via interpolation on the reduced basis space spanned by the set of dominant modes. However, since the reduced dynamics generally belong to nonlinear, manifolds, a variety of interpolation and regression methods have been proposed, capable of enforcing the constraints characterizing those manifolds. Some of the methods most often employed are dynamic mode decomposition [7–9], radial basis function interpolation [10, 11] and Gaussian process regression [12, 13]. The recent advancements in machine learning (ML) methods [14] have given rise to revolutionary approaches that effectively evaluate and expedite existing numerical models or solvers by using online-offline computational stages. In the offline stage, the ML model updates its weights or coefficients (training) to learn the system dynamics by using the high-fidelity solutions obtained by the numerical solver, hence requiring computational power and time. In the online stage, the model uses the pre-computed/optimized weights (from the training) to obtain the solution (prediction) for a new set of input instances and does so almost instantly with minimal computational cost. Various data-driven ML-based frameworks have been proposed to model the propagation of system dynamics in latent space. Some of the more highly successful examples involve the use of deep neural networks (DNNs) [15], long-short-term memory (LSTM) networks [16–19], neural ordinary differential equations (NODE) [19–21], and temporal convolutional networks (TCNs) [22, 23].

Significant work has been carried out recently on predicting solution instances outside the training domain for a variety of fluid problems with discontinuities, wave propagation, and advection-dominated flows. Liu et al. [24] presented a predictive data assimilation framework based on the Ensemble Kalman Filter (EnKF) and the DDROM model, which uses an autoencoder network for the compression of high-dimensional dynamics to lower dimensional space and then the LSTM method to model the fluid dynamics in the latent space. The model capabilities were estimated using 2D Burgers’ equation and flow past a cylinder test case. Maulik et al. [25] proposed a Convolutional Autoencoder (CAE) for compression and a recurrent LSTM network for the time evolution on the reduced space. The CAE-LSTM model was capable of reconstructing the sharp profile of the advecting Burgers’ equation more accurately than the POD-Galerkin technique. Dutta et al. [18] utilized an advection-aware (AA) autoencoder network that learns nonlinear embeddings of the high-fidelity system snapshots using an arbitrary snapshot from the dataset, and then models the latent space dynamics using LSTM network to make predictions for the linear advection and Burgers’ problem. Cheng et al. [26] used the POD-ANN model, in which they performed a priori dimension reduction on the high-fidelity dataset and parameterization with an artificial neural (ANN) network to solve the strongly non-linear Allen-Cahn equations and the cylinder flow problem. Heaney et al. [27] proposed an AI-DDNIROM framework, capable of making predictions for spatial domains, significantly larger than the training domain, using a domain decomposition approach, an autoencoder network for low-rank representation, and an adversarial network for making the predictions for flow past a cylinder and slug flow problems. Fatone et al. [19] introduced a $\upmu$t-POD-LSTM ROM framework that is capable of extrapolation for time windows around 15% those of the training domain on unsteady advection–diffusion and unsteady Navier–Stokes equation for new parameter instances. Xu et al. [23] proposed a multi-level framework comprising a convolution autoencoder (CAE), a temporal CAE (TCAE), and a multilayer perceptron (MLP), for the purpose of parameterization, and a TCN network for auto-regressive future state predictions, and evaluated the results on problems such as Sod’s-shock tube and transient ship waves. Wu et al. [22] developed a POD and TCN-based neural network for making predictions on the viscous periodic flow past a cylinder case. Abdedou et al. [28] proposed two CAE architectures to compress the high-dimensional snapshot matrices obtained from numerical solvers for the Burgers’, Stoker’s, and shallow-water equations in space and time and performed parameterization on the compressed latent space. Jacquier et al. [29] employed uncertainty quantification methods—Deep Ensembles and Variational Inference-based Bayesian Neural Networks on the POD-ANN order-reduction method to perform predictions within and outside of the training domain on problems such as shallow water equations for flood prediction, and generated probabilistic flooding maps aware of model uncertainty. Geneva et al. [30] presented a physics-constrained Bayesian auto-regressive CAE network that models non-linear dynamical systems (Kuramoto–Sivashinsky equation, 1D Burgers’, 2D Burgers’) devoid of training data, using only the initial conditions. This reduces the computation cost tremendously and provides uncertainty quantification at each time step.

The caveat that remains is a long-term temporal extrapolation for fluid problems marked by sharp gradients and discontinuities. Our study explores forecasting convolutional architectures (LSTM, TCN, and CNN) to obtain accurate solutions for time steps distant from the training domain, on advection-dominated test cases. The high-dimensional input snapshots matrix is first compressed in space to obtain the reduced latent vectors before they are passed as a sequence to the forecasting models. Two types of architectures are first evaluated for space compression—MLP autoencoder and CAE autoencoder, to identify the one that is more accurate in terms of the reconstruction and preservation of the input information. A simple convolutional architecture is then proposed and shown to provide accurate results for the forecasts.

The subsequent sections of the paper are organized as follows. “Methodology” section describes the dataset structure along with the training and testing strategies, followed by a presentation of the autoencoders for space compression and the forecasting convolutional architectures. In “Results and discussion” section, the models are tested on three numerical cases which are representative of advection-dominated flows—one-dimensional Burgers’ problem, one-dimensional Stoker’s equations, and two-dimensional shallow-water equations to model a dam-break scenario on a real river. Finally, “Conclusion” section presents a summary of the results obtained by the models and some concluding remarks.

## Methodology

### Dataset

**Fig. 1:**
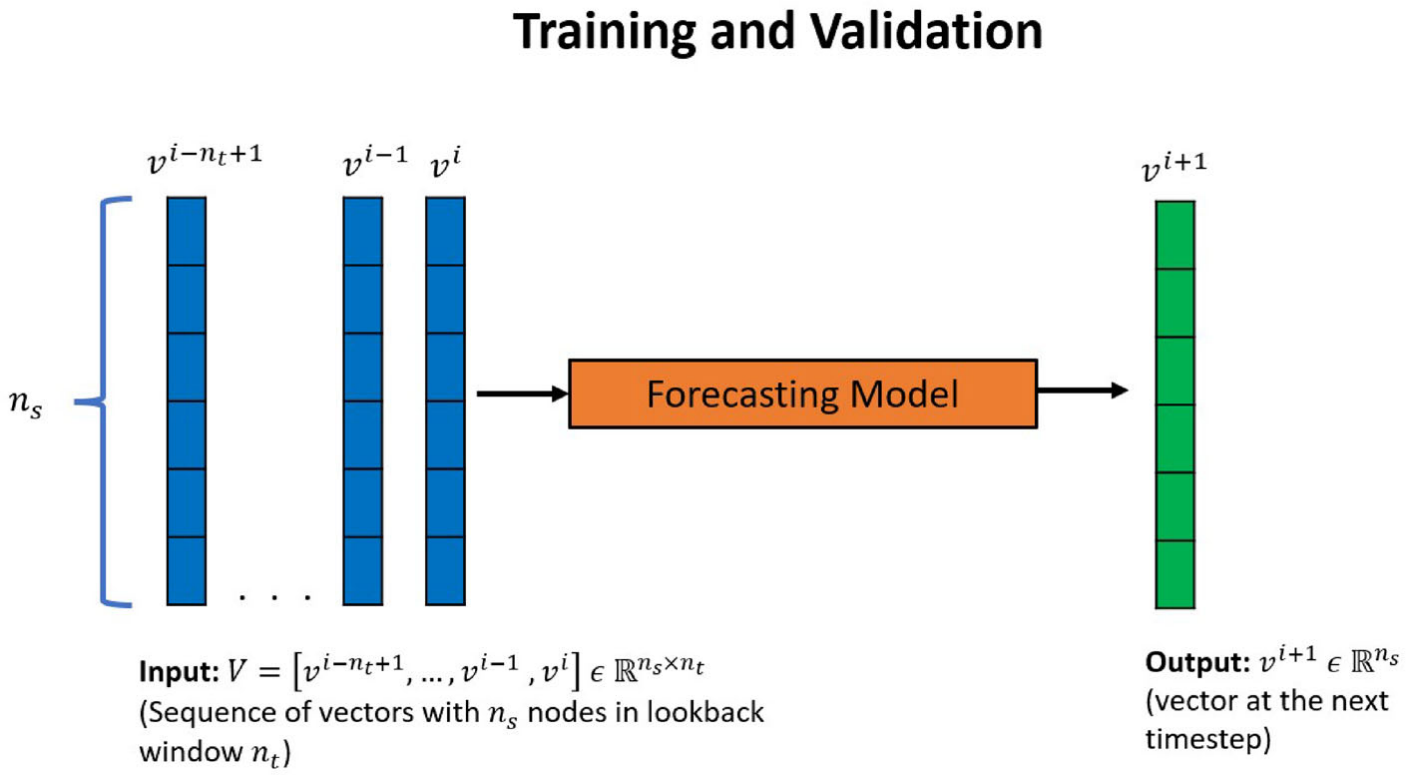
Training and validation method

The dataset is comprised of *T* solution vectors/snapshots: $v^{i}$ with $n_s$ nodes ($v^i \, \in \, {\mathbb {R}}^{n_s}$) at time-steps *i* $\in$ $\{1, 2,..., T\}$ obtained using a high-fidelity PDE solver. For the autoencoder models, the output is the reconstruction of the input, therefore the training and validation input and output data are snapshot vectors $v^{i}$. For the forecasting models (Fig. 1), N samples are used for training; in each sample, the input is a sequence of $n_{t}$ snapshots (lookback window = $n_t$): $V = [v^{i-n_{t}+1},..., v^{i-1}, v^{i}]$, with $V \, \in \, {\mathbb {R}}^{n_s \times n_t}$, and the corresponding output is the vector at the time-step immediately after the sequence end—$v^{i+1}\, \in \, {\mathbb {R}}^{n_s}$.

**Fig. 2:**
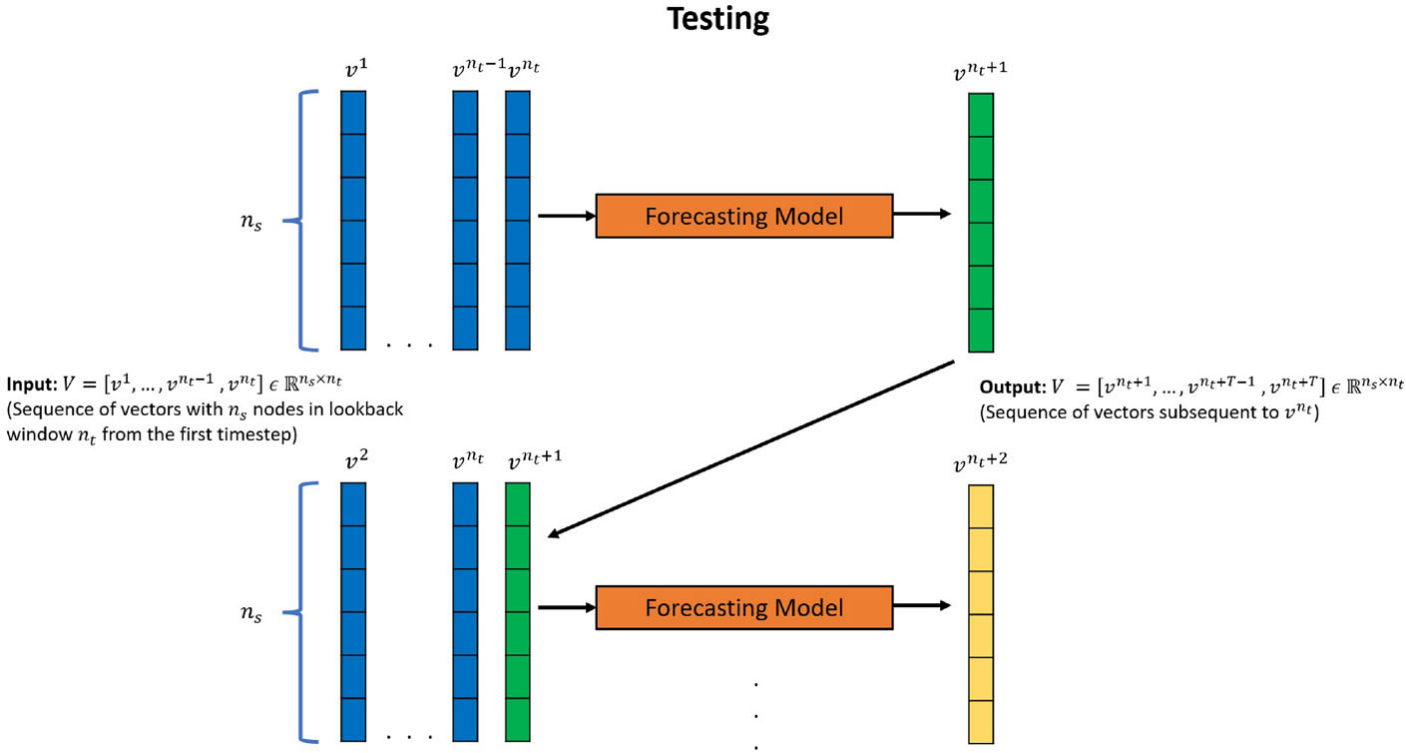
Autoregressive testing method for forecasting models

For extrapolative testing (Fig. 2), a sequence of $n_{t}$ vectors from the start of the dataset, $V = [v^{1},...,v^{n_t-1}, v^{n_{t}}]\, \in \, {\mathbb {R}}^{n_s \times n_t}$ is fed to the model to produce the vectors at all the subsequent time-steps: $[v^{n_{t}+1}, v^{n_{t}+2},..., v^{T}]\, \in \, {\mathbb {R}}^{n_s \times (T-n_t)}$ in an auto-regressive manner, i.e, first only a single subsequent snapshot $v^{n_{t}+1}$ is predicted, which is then concatenated with previous $n_t-1$ vectors and passed to the forecasting model to produce vector $v^{n_{t}+2}$. This process is repeated in accordance with the desired number of subsequent solution vectors.

### Non-intrusive reduced-order modeling

Non-intrusive ROMs (NIROMs) bypass the governing equations and utilize the full-order model solutions to develop a data-driven model, which compresses the full-order data (snapshot) into a reduced-order (latent) space. The method most widely adopted to perform this utilizes deep neural network architectures called autoencoders [31].

**Fig. 3:**
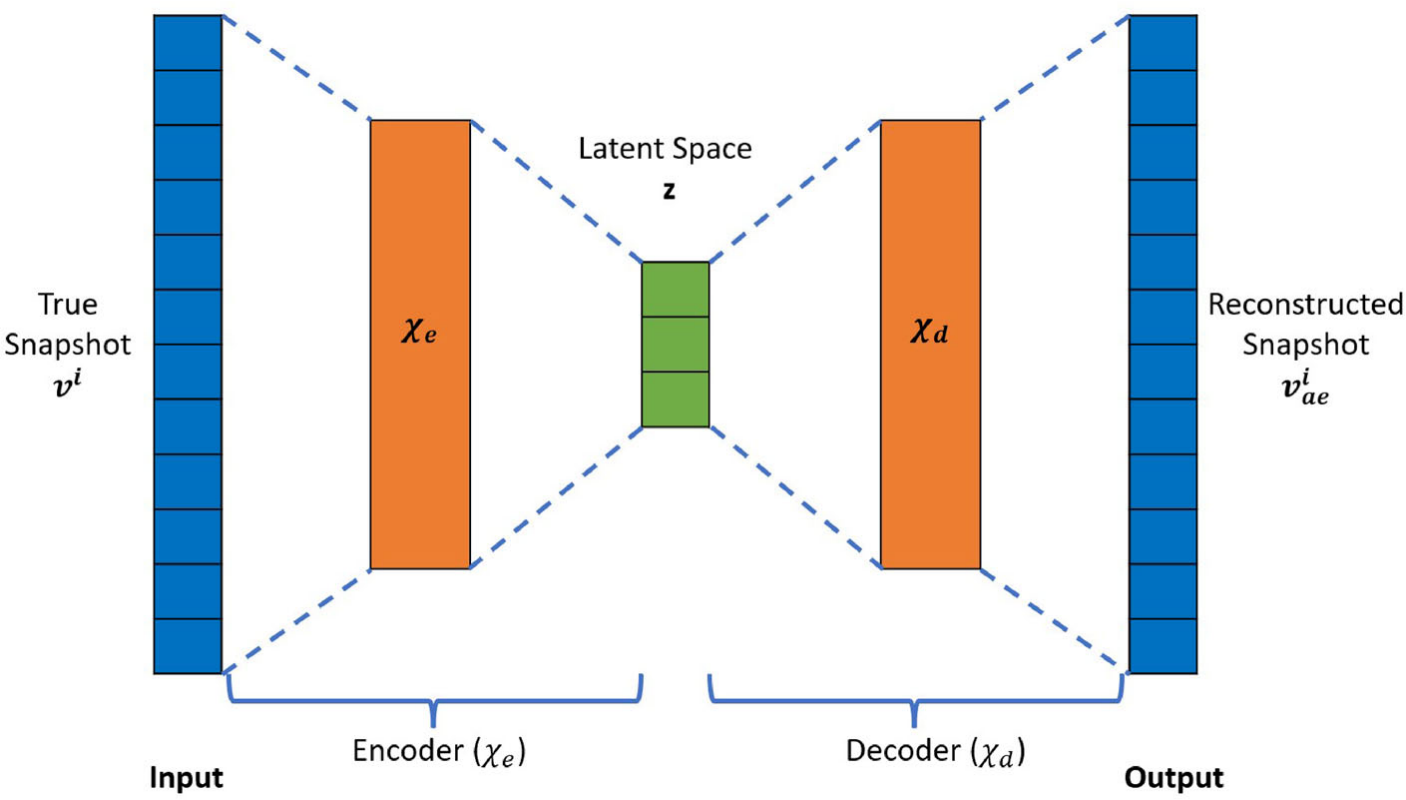
Autoencoder architecture

**Fig. 4:**
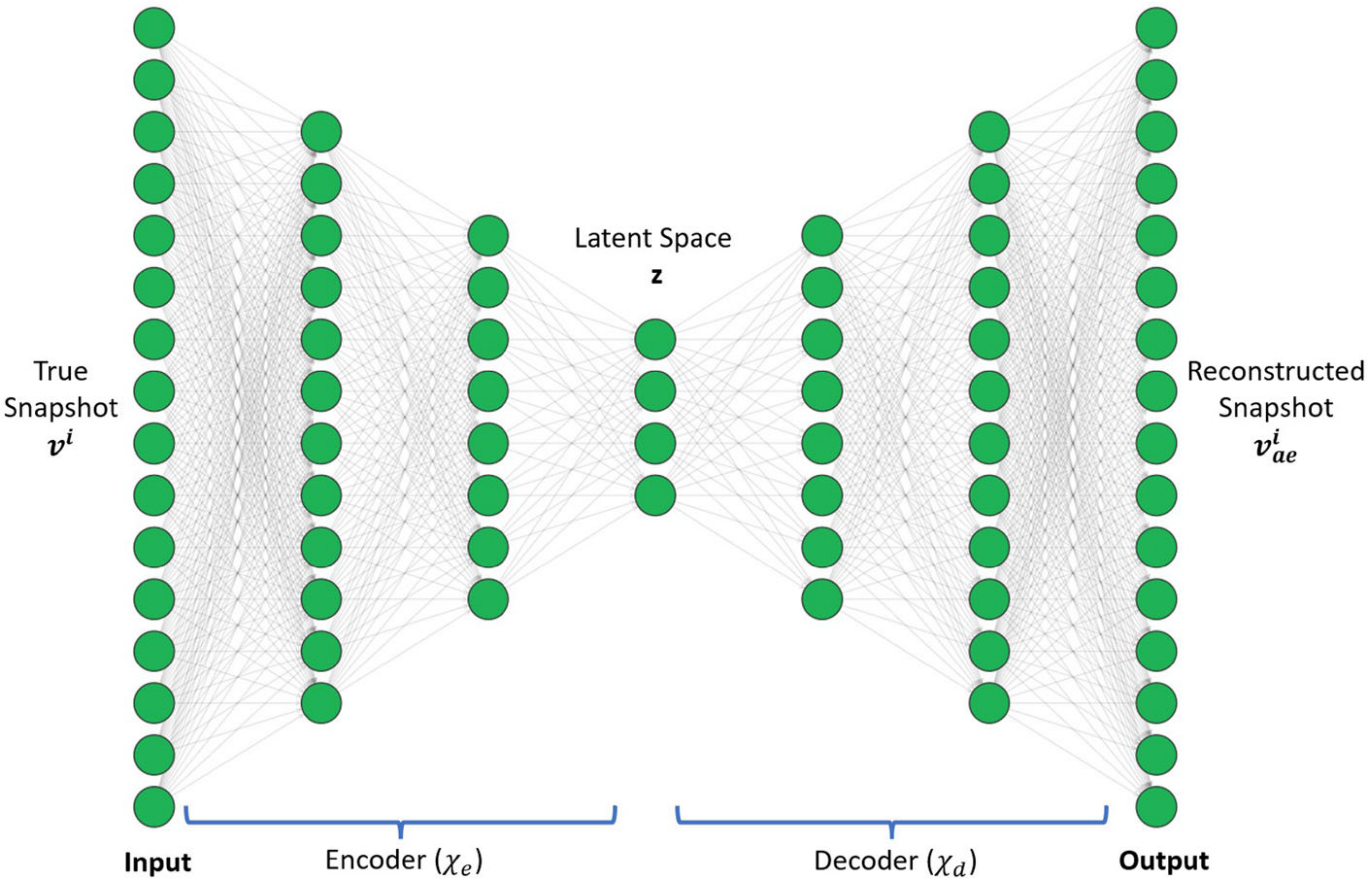
MLP autoencoder architecture

**Fig. 5:**
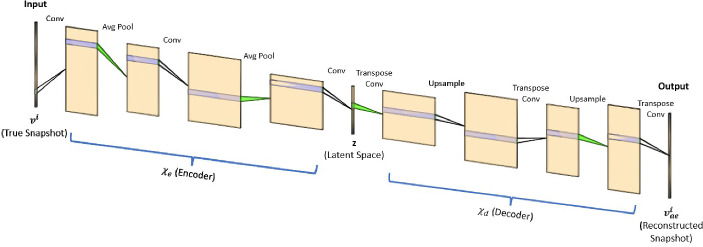
Convolutional autoencoder architecture

An autoencoder learns the approximation of the identity mapping, $\chi$: $v^i \rightarrow v_{ae}^i$ such that $v^i \approx v_{ae}^i$ and $\chi$: ${\mathbb {R}}^{n_s} \rightarrow {\mathbb {R}}^{n_s}$, where ${n_s}$ is the number of nodes in the solution vector $v^i$. This process is accomplished using a two-part architecture. The first part of the autoencoder network is the encoder $\chi _e$, which maps a high-dimensional input vector $v^i$ to a low-dimensional latent vector $z^i$: $z^i = \chi _e (v^i; \theta _e )$ and $z^i$ $\in$ ${\mathbb {R}}^m$ $(m \ll n_s)$. The second part is called a decoder, $\chi _d$, which maps the latent vector $z^i$ to an approximation $v_{ae}^i$ of the high-dimensional input vector $v^i$: $v_{ae}^i$ = $\chi _d (z^i; \theta _d$). The combination of these two parts yields an autoencoder network (Fig. 3) of the form $\chi$: $v^i \rightarrow \chi _d \circ \chi _e(v^i)$. The autoencoder model is trained by computing optimal values of the parameters ($\theta _e$, $\theta _d$) that minimize the reconstruction error over all the training data [18]:

1 $$\begin{aligned} \theta _e, \theta _d = argmin {\mathcal {L}}(v^i, v_{ae}^i) \end{aligned}$$

where ${\mathcal {L}}(v^i, v_{ae}^i)$ is a chosen measure of discrepancy between $v^i$ and its approximation $v_{ae}^i$. The restriction (dim($z^i$) = m) $\ll$ (n = dim($v^i$)) forces the autoencoder model to learn the salient features of the input data via compression into a low-dimensional space and to then reconstruct the input, instead of directly learning the identity function. Autoencoder architectures are generally comprised of MLPs (called AAs) [18], convolutional neural network autoencoders (called CAEs) [23, 25, 28], or a combination of both. While small-sized problems can be effectively modeled via an MLP architecture, problems involving data of high spatial complexity require CAE autoencoders for effective and accelerated spatial compression. The architecture of an MLP autoencoder, with two fully connected dense layers (hidden layers) in the encoder network and a mirrored decoder network, is shown in (Fig. 4). The Convolution autoencoder consists of two convolution layers, each followed by batch normalization, swish activation, and an average pooling layer, as described in (Fig. 5).

### Forecasting techniques

The dataset “Dataset” post compression by the encoder ($\chi _e$) produces *N* samples of the form: $Z=[z^{i-n_{t}+1},..., z^{i-1}, z^{i}] \, \in \, {\mathbb {R}}^{m \times n_t}, \, z^{i+1} \in \, {\mathbb {R}}^m$, which are used to train the following forecasting models.

#### Long short-term memory (LSTM)

LSTM [32] is a special type of recurrent neural network (RNN) that is well-suited for performing regression tasks based on time series data. The main difference between the traditional RNN and the LSTM architecture is the capability of an LSTM memory cell to retain information over time and an internal gating mechanism that regulates the flow of information in and out of the memory cell [33]. The LSTM cell consists of three parts, also known as gates, that have specific functions. The first part called the forget gate, chooses whether the information from the previous step in the sequence is to be remembered or can be forgotten. The second part called the input gate, tries to learn new information from the current input to this cell. The third and final part, called the output gate, passes the updated information from the current step to the next step in the sequence. The basic LSTM equations for an input vector $v^i$ are:

2 $$\begin{aligned} input\,gate: \zeta _{in} = \alpha _s \circ F_{in}(v^i) \end{aligned}$$

3 $$\begin{aligned} forget\,gate: \zeta _{for} = \alpha _s \circ F_{for}(v^i) \end{aligned}$$

4 $$\begin{aligned} cell\,state: c_i = \zeta _{for} \odot c_{i-1} + \zeta _{in}\odot (\alpha _t \circ F_a(v^i)) \end{aligned}$$

5 $$\begin{aligned} output\,gate: \zeta _{out} = \alpha _s \circ F_{out}(v^i) \end{aligned}$$

6 $$\begin{aligned} output: h_i = \zeta _{out}\circ \alpha _t(c_i) \end{aligned}$$

**Fig. 6:**
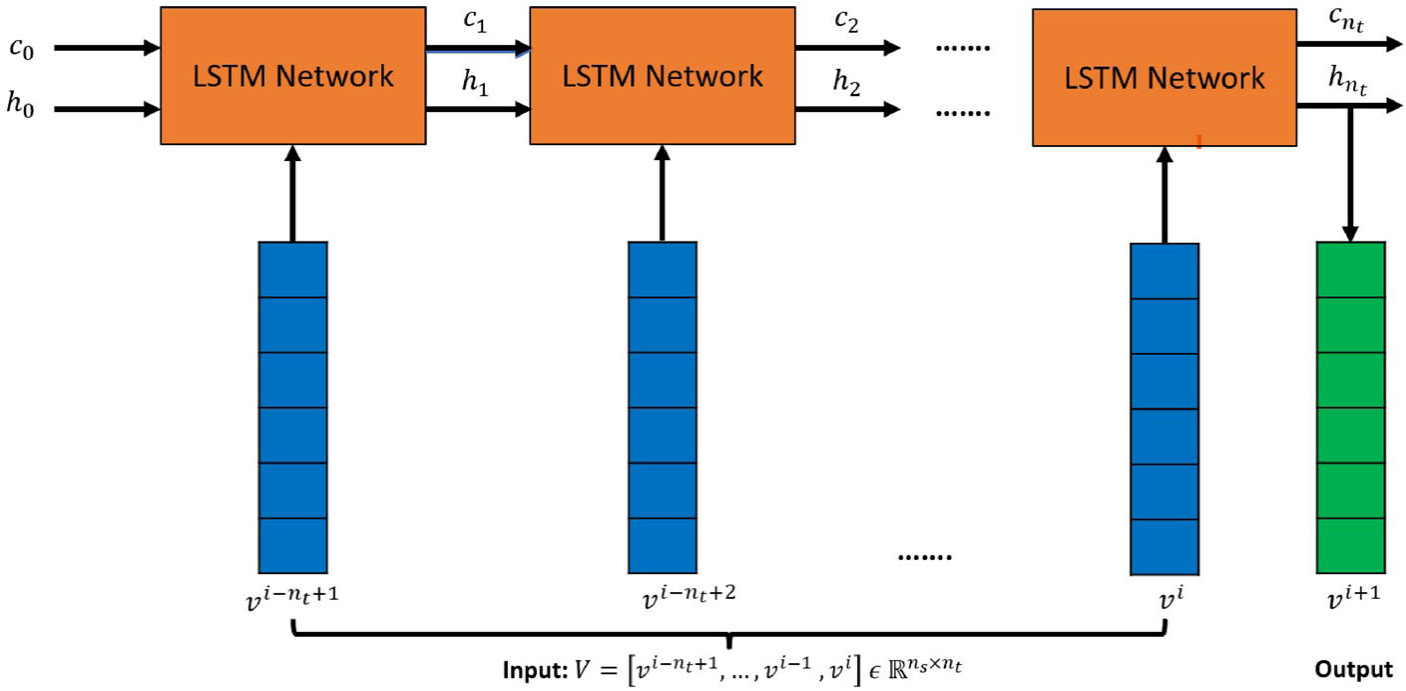
LSTM recurrence on vector sequence

Here, *F* refers to a linear transformation defined by a matrix multiplication and bias addition, that is, $$ F(v^i) = W v^i + b$$, where W $$\in $$ $${\mathbb {R}}^{h \times n_s}$$ is a matrix of layer weights (*h* is number of neurons in the LSTM cell), b $$\in $$ $${\mathbb {R}}^{h}$$ is a vector of bias values, and $$v^i$$ $$\in $$ $${\mathbb {R}}^{n_s}$$ is the input vector to the LSTM Cell. Also, $$\alpha _s$$ and $$\alpha _t$$ denote sigmoid and hyperbolic tangent activation functions, respectively, which are standard choices in an LSTM network, and $$x \odot y$$ denotes a Hadamard product of two vectors *x* and *y*. The sequence of snapshot vectors of $$n_{t}$$ time-steps: $$V = [v^{i-n_{t}+1},..., v^{i-1}, v^{i}]$$, with $$V \, \in \, {\mathbb {R}}^{n_s \times n_t}$$ trains the LSTM network, with recurrence over time (Fig. 6), to predict the subsequent vector $$v^{i+1}$$. The core concept of an LSTM network is the cell state $$c_i$$, which behaves as the “memory” of the network. It can either allow greater preservation of past information, reducing the issues of short-term memory, or it can suppress the influence of the past, depending on the actions of the various gates during the training process.

#### Temporal Convolution Network (TCN)

**Fig. 7:**
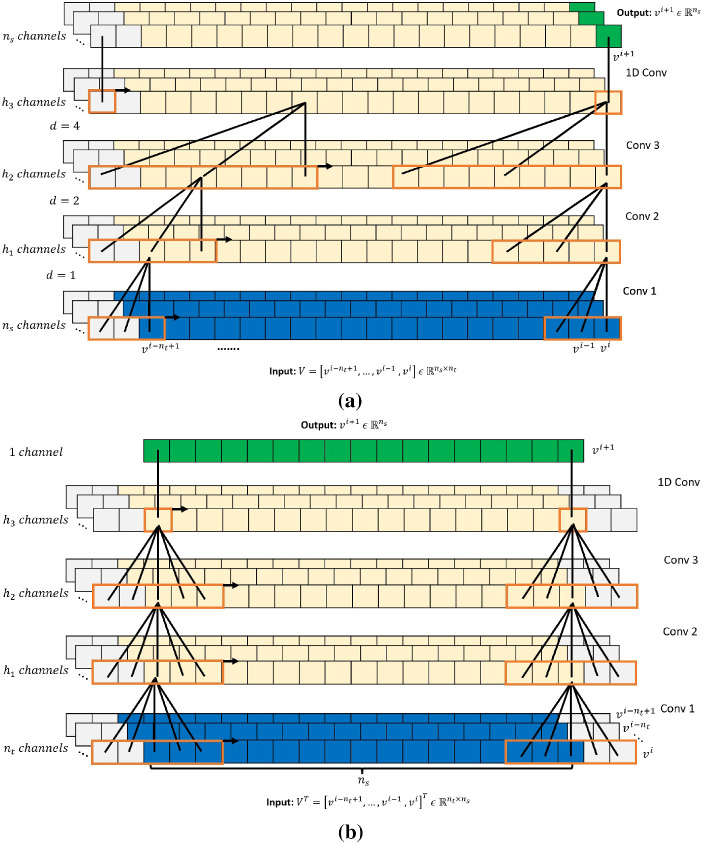
**a** 1D dilated filters convolving on the temporal dimension of vectors, **b** 1D CNN filters convolving on the spatial dimension of vectors

The TCN is based on two principles [34]: the network produces an output of the same length as the input, and there can be no leakage from the future into the past. To verify that the first principle is respected, the TCN uses a 1D fully-convolutional network (FCN) where each hidden layer has the same length as the input layer, and zero padding of length $(k -1)$ is added to keep subsequent layers the same length as previous ones. To respect the second principle, the TCN uses causal convolutions (achieved by padding only on the starting side of input sequences), where the output at time *i* is convolved only with elements from time *i* and earlier in the previous layer (Fig. 7). A TCN also makes use of dilated convolutions that enable an exponentially large receptive field. For an input sequence, $V \ \in$ ${\mathbb {R}}^{n_s \times n_t}$ and a kernel *K* with learnable weights, $K \ \in \ {\mathbb {R}}^k$ (*k* is the kernel size), the element *O*(*s*) with s $\in \{0, 1,..., n_t-k+1\}$ produced by the dilated 1D convolution is:

7 $$\begin{aligned} O(s) = \sum _{j=0}^{k-1}V(s+j*d) \times K(j) \end{aligned}$$

where *d* is the dilation factor and *k* is the kernel size. When using dilated convolutions, *d* is increased exponentially with the depth of the network (eg., $d = 2^l$ at level *l* of the network), ensuring that some filter hits each input within a large effective history.

In the TCN model employed here, a generic residual block is used in place of a convolutional layer. A residual block contains a branch leading to a series of transformations obtained by layers of TCNs, whose outputs are added to the input *V* of the block to obtain $O_{rb}$:

8 $$\begin{aligned} O_{rb} = Activation(V + F(V)) \end{aligned}$$

**Fig. 8:**
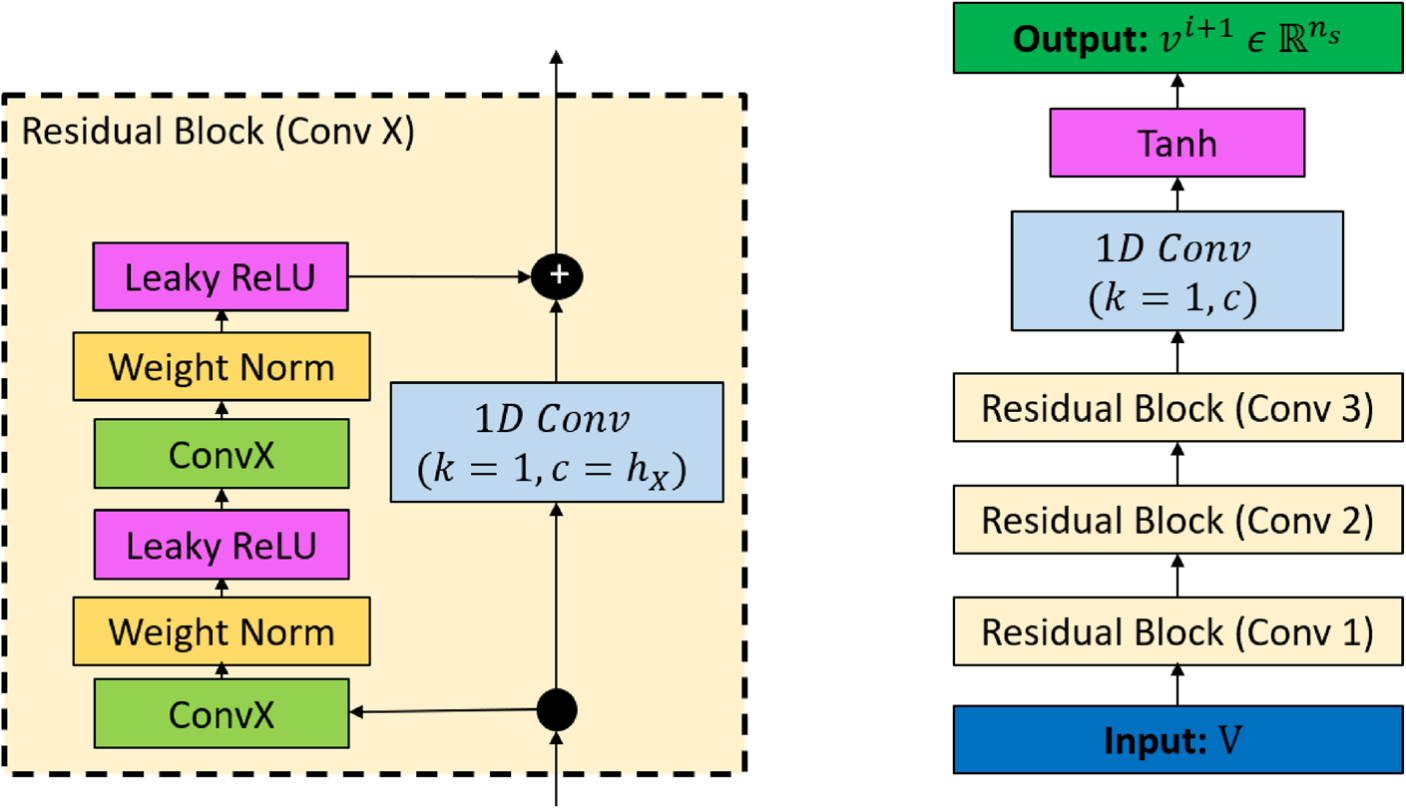
Residual Block (left) and architecture of CNN and TCN (right). For TCN, the input vector $V \ \in$ ${\mathbb {R}}^{n_s \times n_t}$ and $c = n_{s}$. For CNN, the input vector $V \ \in$ ${\mathbb {R}}^{n_t \times n_s}$ and $c = 1$

Within a residual block (Fig. 8), the TCN has two layers of dilated causal convolution with weight normalization and non-linearity, with a leaky rectified linear unit (leaky ReLU). To account for different input–output widths during addition operations, a 1D convolution (kernel size = 1 and channels = $n_s$) is used to ensure the element-wise addition operator ($\oplus$) receives tensors of the same shape.

When convolving along the temporal axis, this (*standard*) TCN model uses information available from all the prior time steps (due to the large receptive field) to evaluate the next time step, as sketched in Fig. 7. The model takes in a sequence of $n_{t}$ vectors corresponding to a look-back window of size $n_{t}$: $V = [v^{i-n_{t}+1},..., v^{i-1}, v^{i}]$, with $V \, \in \, {\mathbb {R}}^{n_s \times n_t}$. The filters convolve along the temporal axis for all the $n_s$ vector nodes since the nodes are passed in as channels. However, the results produced from this model “Results and discussion” section do not propagate beyond the training domain. Therefore, another model is proposed here, where the dilated convolutions of the TCN model convolve along the spatial axis and thus use the information available from the neighboring nodes to determine the future time-step value of the node. This model takes in a sequence of $n_{t}$ vectors corresponding to a look-back window of size $n_{t}$ in a transposed manner, such that the $n_{t}$ solution vectors are on separate channels: $V^T \, \in \, {\mathbb {R}}^{n_t \times n_s}$, where $V = [v^{i-n_{t}+1},..., v^{i-1}, v^{i}]$. This model produces significantly better results than the TCN on a temporal axis, but the causal padding and dilations employed are of no significance when the convolution filter operates along the spatial axis. Another architecture for modeling the system dynamics, with 1D convolutions and without any dilations or causal paddings, is therefore proposed in the following section.

####  A proposed Convolution Neural Network (CNN) for time forecasting

A convolutional layer convolves filters with trainable weights on the input vector $v^i$ [31]. Such filters are commonly referred to as convolutional kernels. In a convolutional neural network, the inputs and outputs can have multiple channels. For a convolutional layer with $n_{in}$ input channels and $n_{out}$ output channels, the total number of convolutional kernels is $n_{k} = n_{i} \times n_{o}$. Each kernel slides along the spatial direction and the products of kernel weights and vector nodes are computed at all sliding steps. For an input vector $v^i$ and a kernel *K*, the corresponding output feature map *O*(*s*) with s $\in \{0, 1,..., n_s-k+1\}$ (where *k* is the kernel size) is given by:

9 $$\begin{aligned} O(s) = \sum _{j=0}^{k-1}v^i(s+j) \times K(j) \end{aligned}$$

Zero padding of size $(k-1)/2$ is added to both sides of the output feature map to maintain the spatial dimension as $n_s$.

The proposed forecasting model of CNN takes in a sequence of vectors with $n_{t}$ time-steps in a transposed manner as its input: $V^T \, \in \, {\mathbb {R}}^{n_t \times n_s}$, where $V = [v^{i-n_{t}+1},..., v^{i-1}, v^{i}]$, so that the filter convolves on *the spatial dimension* of size $n_s$, and the $n_{t}$ vectors lie on separate channels, as shown in Fig. 7. The CNN architecture (Fig. 8) consists of *X* residual blocks (*X* is a hyperparameter), in which the input to each block, after transformation (to make the channels equal) from a 1D Convolution layer (kernel = 1 and channels = 1) is added to the output from the block. A residual block consists of two convolution layers, each followed by a weight normalization and a leaky ReLU activation layer.

#### Metrics

To evaluate the performance of the previous architectures, the following metrics are used:

*Mean Squared Error* ($L_2$ *norm*): The average of the square of the difference between the actual $v_i$ and predicted values ${\hat{v}}_i$ over *N* samples:

10 $$\begin{aligned} MSE = \frac{\sum _{i=1}^N(v_{i}-{\hat{v}}_{i})^2}{N} \end{aligned}$$

*Mean Absolute Error* ($L_1$ *norm*): The average of the difference between the two vectors $v_i$ and ${\hat{v}}_i$ over *N* samples:

11 $$\begin{aligned} MAE = \frac{\sum _{i=1}^N \Vert v_{i}-{\hat{v}}_{i}\Vert }{N} \end{aligned}$$

*Relative* $L_2$ *Norm Error*: The relative $L_2$ norm error (referred as error) is calculated as:

12 $$\begin{aligned} Relative Error = \frac{\sqrt{\sum _{i=1}^N (v_{i}-{\hat{v}}_{i})^2}}{\sqrt{\sum _{i=1}^N v_{i}^2}} \end{aligned}$$

## Results and discussion

The capability of the autoencoders (MLP-AE and CAE) to efficiently transform high-dimensional vectors to a low-dimensional space, and that of the forecasting models (LSTM, TCN, and CNN) to accurately model the system dynamics were tested using advection-dominated flow problems.

### 1D Burgers’ problem

The test case involves the one-dimensional Burgers’ equation, which is a non-linear advection–diffusion PDE. The equation along with the initial and Dirichlet boundary conditions are given by

13 $$\begin{aligned} \frac{\partial u}{\partial t} + u\frac{\partial u}{\partial x} = \nu \frac{\partial ^2 u}{\partial t^2} \end{aligned}$$

14 $$\begin{aligned} x \in [0, L], u(0, t) = 0 \end{aligned}$$

15 $$\begin{aligned} u(x, 0) \equiv u_0 = \frac{x}{1+\sqrt{\frac{1}{t_0}}\exp ({Re\frac{x^2}{4}})} \end{aligned}$$

where the length $L = 1m$ and the maximum time $T_{max} = 2\,s$. The solutions obtained from the above equations produce sharp gradients even with smooth initial conditions if the viscosity $\nu$ is sufficiently small, due to the advection-dominated behaviour. The analytical solution to the problem is given by:

16 $$\begin{aligned} u(x, t) = \frac{\frac{x}{t+1}}{1+\sqrt{\frac{t+1}{t_0}}\exp ({Re\frac{x^2}{4t+4}})} \end{aligned}$$

where $t_0 = \exp ({\frac{Re}{8}})$ and $Re = 1/\nu$. The high-fidelity solution vectors are generated by directly evaluating the analytical solution over a uniformly discretized spatial domain containing 200 grid points ($n_s = 200$) at 250 uniform time-steps ($T = 250$) for two different values of *Re*: 300 and 600. The solution vectors obtained are then used to train the autoencoder and forecasting models “Dataset” section. For the autoencoder training, 200 solution vectors are chosen at random time steps, and the remaining 50 are used for validation. For the forecasting model, the training set is comprised of the first 150 compressed samples, each sample containing $n_t$ consecutive solution vectors (i.e. look back window = $n_t$), where $n_t$ is a hyperparameter. The validation set consists of subsequent 10 samples. For testing, $n_t$ latent vectors from the start of the dataset are fed to the forecasting model to predict the subsequent time steps via auto-regression (Table 1).

**Table 1:** Burgers’ problem: training, validation and testing dataset

**Dataset**	**Samples**	**Input**	**Output**
Training	1	$[z^{1},..., z^{n_t-1}, z^{n_t}]$	$z^{n_t+1}$
	2	$[z^{2},..., z^{n_t}, z^{n_t+1}]$	$z^{n_t+2}$
	...	...	...
	150	$[z^{150},..., z^{n_t+148}, z^{n_t+149}]$	$z^{n_t+150}$ (training end)
Validation	151	$[z^{151},..., z^{n_t+149}, z^{n_t+150}]$	$z^{n_t+151}$
	...	...	...
	160	$[z^{160},..., z^{n_t+158}, z^{n_t+159}]$	$z^{n_t+160}$
Testing	1	$[z^{1},..., z^{n_t-1}, z^{n_t}]$	$[z^{n_t+1},..., z^{249}, z^{250}]$

#### Autoencoders for spatial compression

Two types of autoencoder architectures “Non-intrusive reduced-order modeling” section—MLP (referred to as AE) and Convolutional (referred to as CAE) are proposed for the compression of solution vectors—$v^i \, \in {\mathbb {R}}^{n_s}$ to latent vectors $z^i \, \in {\mathbb {R}}^{m}$ by the encoder $\chi _e$. Sequences formed from these latent vectors ($[z^{i-n_{t}+1},..., z^{i-1}, z^{i}] \, \in \, {\mathbb {R}}^{m \times n_t}$) are utilized to train the forecasting models—LSTM, TCN and CNN. The trained models are then used to forecast the latent vectors at subsequent time steps to the sequence ($[z^{n_{t}+1}, z^{n_{t}+2},..., z^{T}] \, \in \, {\mathbb {R}}^{m \times (T-n_t)}$) given as input to the forecasting model. The latent vectors are then reconstructed into solution vectors ($[v^{n_{t}+1}, v^{n_{t}+2},..., v^{T}] \, \in \, {\mathbb {R}}^{n_s \times (T-n_t)}$) using the decoder $\chi _d$. The heat map plots obtained by stacking these reconstructed solution vectors along the x-axis (spatial nodes-$n_s$ along y, time-steps-$n_t$ along x), are illustrated for both autoencoder models in Appendix: Table 14). Separate compression and forecasting models were used for cases—Re = 300 and Re = 600.

Both architectures, AE and CAE, are capable of efficiently compressing the solution vectors to latent vectors with few modes and fine reconstruction/decompression. However, only CAE compression followed by CNN autoregression produces accurate results on extrapolation. This is because the proposed CAE architecture is devoid of any dense layer (single layer of neurons), and therefore even during compression, the local spatial information in the vector remains preserved. This consistency facilitates the modeling of latent dynamics by the CNN model, as it convolves on the spatial axis of the input and utilizes information from the neighboring cells at the provided time steps to predict nodal values at subsequent time steps.

The hyperparameters for the AE and CAE architectures are listed in Table 2, where encoder layers denote the number of neurons in the two dense encoder layers of AE, and the number of channels in the convolution layers of CAE. The decoders of both autoencoders are mirrored structures of their encoders.

**Table 2:** Burgers’ problem: hyperparameters for the AE and CAE networks

**Hyperparameters**	**MLP AE**	** Convolutional CAE**
Encoder layers	[100, 75]	[8, 32]
Latent dimension (*m*)	10, 25, 50	12, 25, 50
Activation	relu, swish	relu, swish
Loss function	MSE	MSE
Learning rate	$10^{-3},3 \times 10^{-4}$	$10^{-3},3 \times 10^{-4}$
Model parameters ($m = 50$)	57,060	2018

In the subsequent sections, the results of the forecasted model are produced using compression via the CAE, with 2 layers having 8 and 32 channels in the encoder respectively and a Latent dimension of 50. The kernel size of the 1D convolution is 3, each layer has a padding of size 1. All the hidden layers use swish activation.

#### LSTM model

When LSTM “Long short-term memory (LSTM)” section is used as the future step predictor, it takes in a sequence of $n_t$ (lookback window) latent vectors (spatial dimension = *m*) obtained by compression from the encoder network ($Z \, \in \, {\mathbb {R}}^{m \times n_t}$) to produce the latent vector for the next time-step ($z^{i+1}\, \in \, {\mathbb {R}}^{m}$). The LSTM model consists of multiple LSTM layers stacked together, each having a hidden dimension equal to the latent dimension of the solution vectors. Various sets of hyperparameters considered for the LSTM network, for both Re 300 and 600 are summarized in Table 3.

**Table 3:** Burgers’ problem: hyperparameters for the LSTM network

**Hyperparameters**	**Values**
Sequence length ($n_t$)	5, 10, 20
LSTM layers	1, 2, 3
hidden/latent dimension (*m*)	12,25,50
Activation	tanh
Loss function	MSE
Learning rate	$5 \times 10^{-4}$
Max model parameters ($n_t = 10$)	20,400
Min model parameters ($n_t = 10$)	1248

**Fig. 9:**
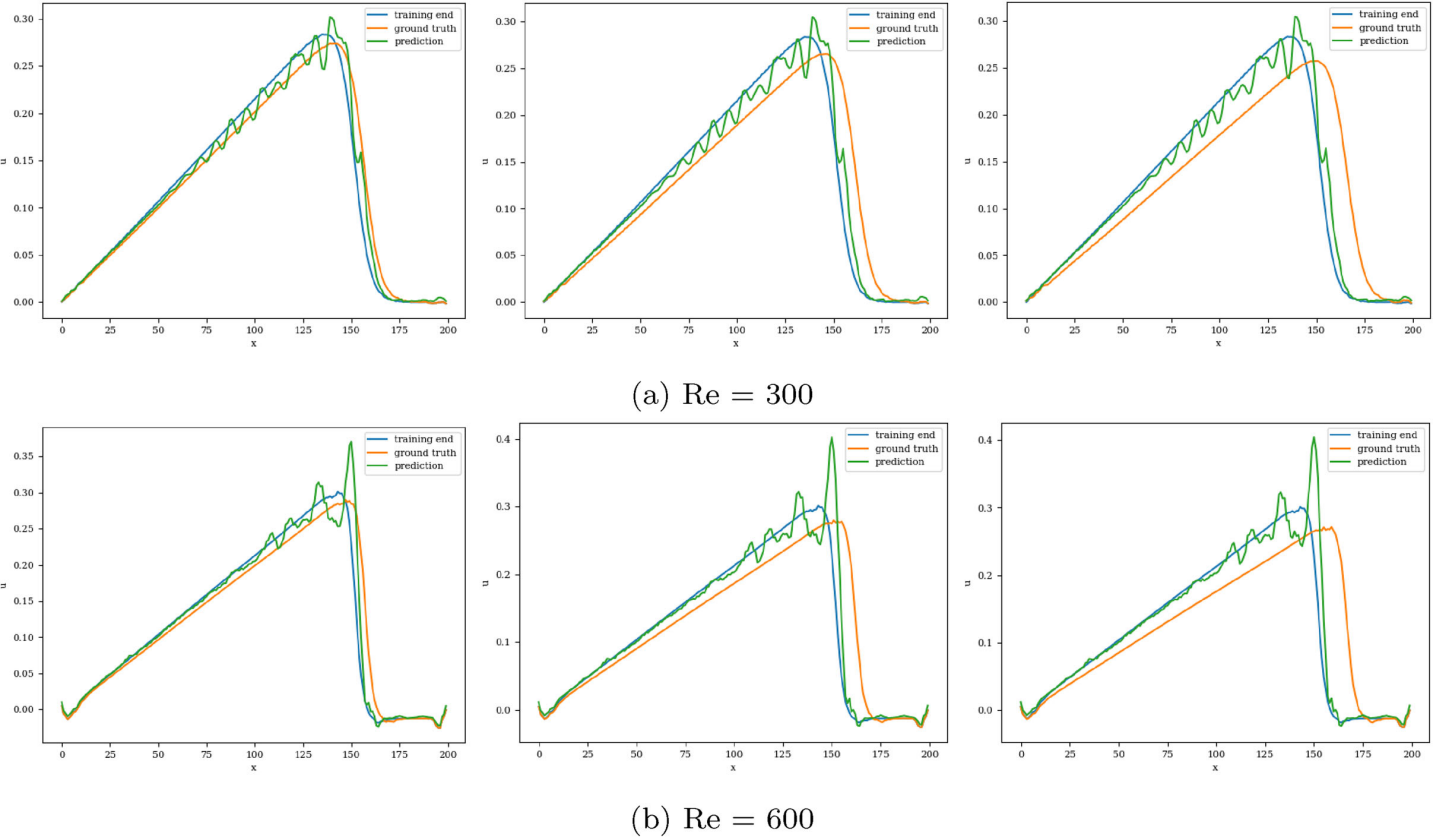
Burgers’ problem: Extrapolative auto-regressive predictions by the LSTM model for time-steps = 180, 200, and 220. The training end time-step = 160; for Re=300 (**a**) and Re= 600 (**b**)

**Fig. 10:**
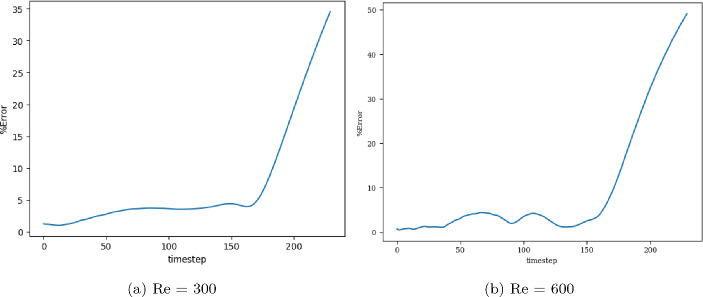
Burgers’ problem: $L_2$ relative error of the autoregressive predictions with increasing time for Re = 300 (**a**) and Re = 600 (**b**) for the LSTM model

The models are trained in batches of size 15, and the loss values for both training and validation converge in 3000 epochs. The model with the least validation loss has a lookback window of size 10 and a single LSTM layer with hidden dimension 50 (latent dimension) for both Re 300 and 600. The extrapolation (Fig. 9) and error plots (Fig. 10) obtained from these models show that the LSTM model accurately predicts the solution vectors for time-steps within the training domain ($i <= 150$), but the solution does not change for time-steps outside the training domain, and so the relative error increases drastically, reaching 35% for Re= 300 and 50% for Re=600 at time-step 200.

#### TCN model

Similar to the LSTM, the TCN model “Temporal Convolution Network (TCN)” section takes in a sequence of $n_t$ latent vectors obtained by compression from the encoder network ($Z \, \in \, {\mathbb {R}}^{m \times n_t}$) to produce the latent vector for the next time-step ($z^{i+1}\, \in \, {\mathbb {R}}^{m}$), with the dilated convolutions operating on the temporal axis, and the latent dimension passed as a channel. The TCN model consists of multiple TCN blocks, each with the same kernel size and number of channels, but with dilations increasing by a factor of 2 in subsequent blocks. The hyperparameters for the TCN network for both Re 300 and 600 are summarized in Table 4.

**Table 4:** Burgers’ problem: hyperparameters for the TCN network

**Hyperparameters**	**Values**
Sequence length ($n_t$)	5, 10, 20
TCN block channels	[32, 32], [64, 64], [32, 32, 32], [64,64,64]
Latent dimension (*m*)	12, 25, 50
Kernel size (k)	3, 5, 7, 9
Activation	tanh
Loss function	MSE
Learning rate	$1 \times 10^{-4}$
Max model parameter ($n_t = 10$, $m = 50$)	78,322
Min model parameters ($n_t = 10$, $m = 50$)	17,554

**Fig. 11:**
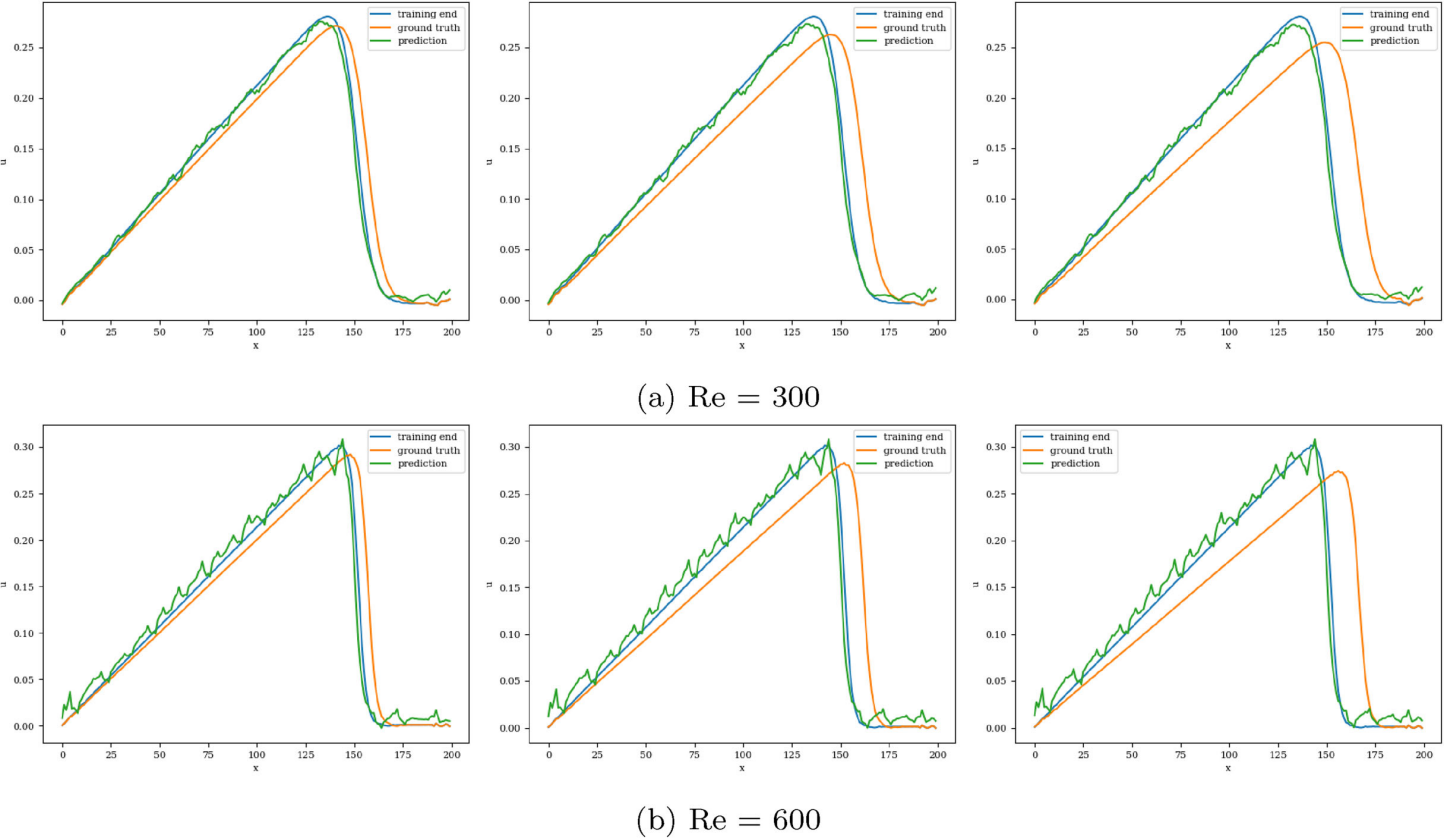
Burgers’ problem: Extrapolative auto-regressive predictions using the TCN model (over time) for Re= 300 (**a**) and Re= 600 (**b**) and for time-steps = 180, 200 and 220; the training end time-step = 160

**Fig. 12:**
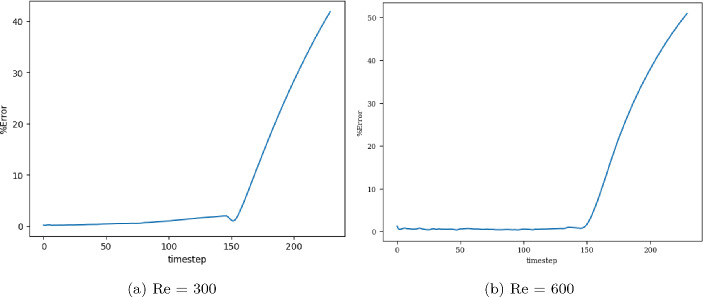
Burgers’ problem, $L_2$ relative error of the auto-regressive predictions with increasing time for Re = 300 (**a**) and Re = 600 (**b**) for the TCN model (over time)

When models are trained in batches of size 15, the training and validation losses reach their minimum values in 4000 epochs. The model with the least validation loss takes in sequence with lookback window 10 and latent dimension 50. For Re 300, the best model has 3 temporal blocks, each having 64 channels, whereas, for Re 600, it has 2 TCN blocks with kernel size 3 and 64 channels each. The extrapolation (Fig. 11) and error plots (Fig. 12) obtained from these models indicate that the TCN model accurately predicts the solution vectors for time steps within the training domain ($i <= 150$), but stops being accurate after the end of training so that the error increases to 40% for Re= 300 and 50% for Re= 600. However, if the same model architecture operates on the input sequence, such that the dilated 1D convolutions propagate along the spatial axis, with each solution vector on a separate channel, then accurate forecasts are produced, even outside the training domain. This encourages the development of a simpler predictive/forecasting model, devoid of dilations and causal padding since the exponentially increasing receptive field serves no purpose when operating along the spatial axis.

#### CNN model

The proposed CNN model “A proposed Convolution Neural Network (CNN) for time forecasting” section takes in a sequence of $n_t$ latent vectors obtained by compression from the encoder network ($Z^T \, \in \, {\mathbb {R}}^{n_t \times m}$) to produce the latent vector for the next time-step ($z^{i+1}\, \in \, {\mathbb {R}}^{m}$), with 1D convolutions operating on the spatial axis (latent dimension) and $n_t$ latent vectors on separate channels. The CNN model consists of two residual blocks, thus compressing the spatial dimension to 50 in the latent vector. Each block possesses the same kernel size and number of channels. The hyperparameters for the CNN network for both Re = 300 and 600 are summarized in Table 5.

**Table 5:** Burgers’ problem: hyperparameters for the CNN network

**Hyperparameters**	**Values**
Sequence length ($n_t$)	5, 10, 20
CNN block channels	[50, 50], [100, 100], [200, 200]
Latent dimension (*m*)	12,25,50
Kernel size (k)	3, 5, 7, 9
Activation	tanh
Loss function	MSE
Learning rate	$1 \times 10^{-4}$
Max model parameters ($n_t = 10$, $m = 50$)	370,001
Min model parameters ($n_t = 10$, $m = 50$)	25,001

**Fig. 13:**
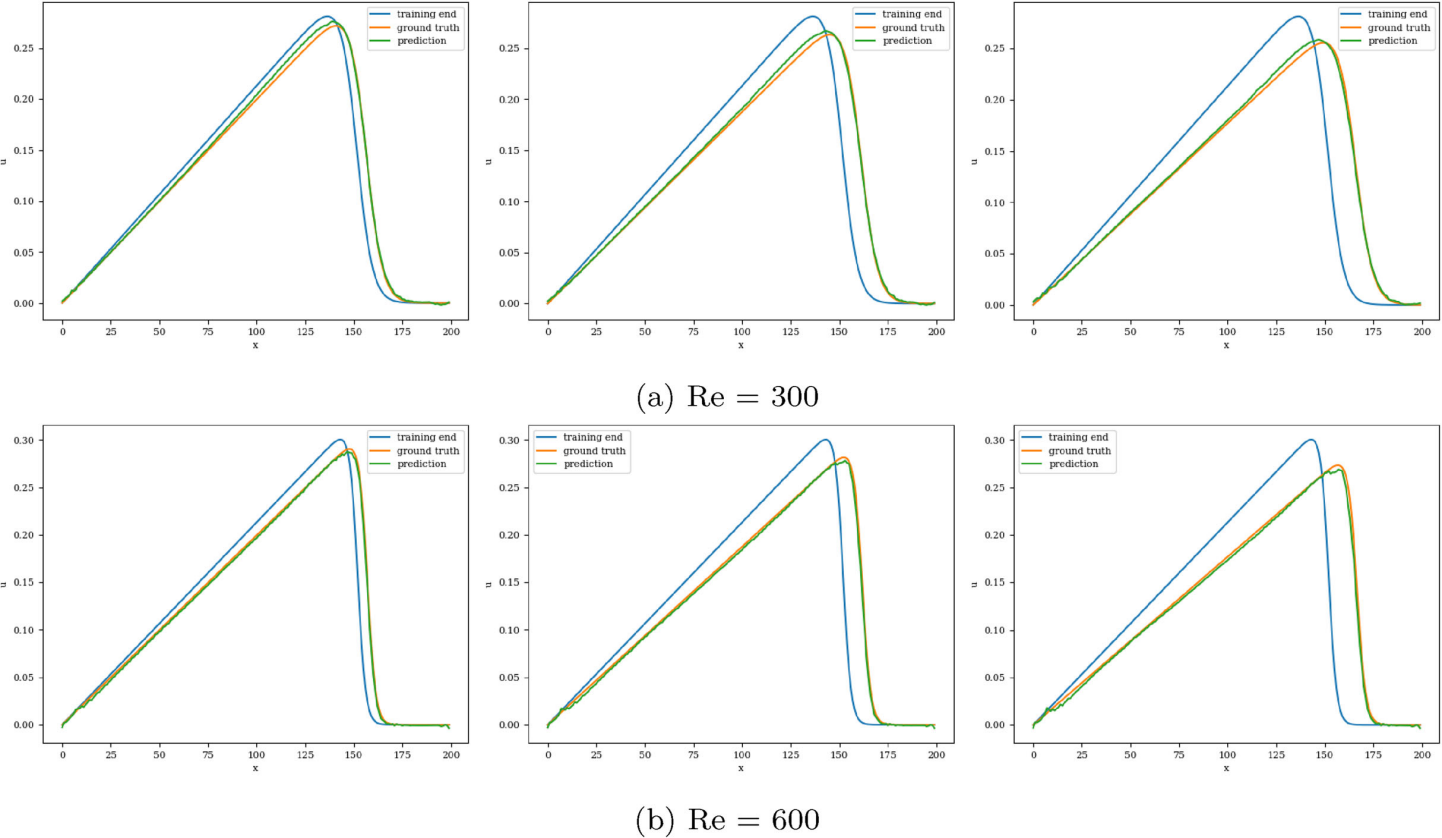
Burgers’ problem: Extrapolative auto-regressive predictions using the proposed CNN model, for Re= 300 (**a**) and Re= 600 (**b**) and for time-steps = 180, 200 and 220; the training end time-step = 160

**Fig. 14:**
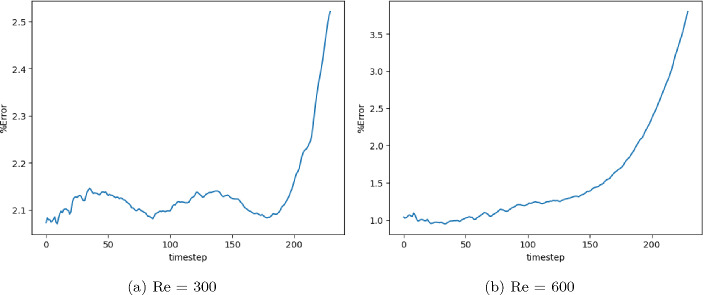
Burgers’ problem: $L_2$ relative error of the auto-regressive predictions using the CNN model with increasing time for Re = 300 (**a**) and Re = 600 (**b**)

The model with the least validation loss has a lookback window of size 10, and each of its blocks has a kernel size of 3 and 50 channels for both Re= 300 and 600. Training and validation loss converges in 3000 epochs for batch size 15. It is clear from the extrapolation (Fig.  13) and error plots (Fig. 14) that the CNN model accurately models the latent dynamics, and predicts solution vectors accurately for time steps beyond the training domain. The error values increase with time, due to the accumulation of errors, since each subsequent time step is predicted auto-regressively; i.e., using previously predicted time steps that contain slight errors. Still, the error reaches a mere 2.5% for Re= 300 and 3.5% for Re= 600, which is significantly less than that produced by other models.

### 1D Stoker’s problem

Stoker’s solution describes the propagation and rarefaction wave resulting from a one-dimensional dam break over a wet, flat, frictionless bottom. Stoker’s problem is considered among the most challenging benchmark test case due to its strong hyperbolic behavior and the discontinuity accompanying the propagation of the front wave resulting from the initial break. The dynamic is initiated by unequal water levels of both the upstream and downstream sides located in the middle of the studied domain of 100 m.

The upstream water level is considered as an input random variable whose values are uniformly sampled within its plausible variability range $h_{up} \, \in \, {U (8, 11)}$, whereas the downstream water depth is kept constant at a deterministic value $h_{ds} \, = \, 1 m$. The analytical solution for the water level is given as:

17 $$\begin{aligned} h(x, t) = {\left\{ \begin{array}{ll} h_{up} &{} \text {if }\, x \le x_A(t)\\ \frac{4}{9g}(\sqrt{gh_{up}-\frac{x}{2t}})^2 &{} \text {if }\, x_A(t) \le x \le x_B(t)\\ \frac{c_m^2}{g} &{} \text {if }\, x_B(t) \le x \le x_C(t)\\ h_{ds} &{} \text {if }\, x_C(t) \le x\\ \end{array}\right. } \end{aligned}$$

**Table 6:** Stoker’s problem: training, validation and testing dataset

**Dataset**	**Samples**	**Input**	**Output**
Training	1	$[z^{1},..., z^{n_t-1}, z^{n_t}]$	$z^{n_t+1}$
	2	$[z^{2},..., z^{n_t}, z^{n_t+1}]$	$z^{n_t+2}$
	...	...	...
	250	$[z^{250},..., z^{n_t+248}, z^{n_t+249}]$	$z^{n_t+250}$ (training end)
Validation	251	$[z^{251},..., z^{n_t+249}, z^{n_t+250}]$	$z^{n_t+251}$
	...	...	...
	260	$[z^{260},..., z^{n_t+258}, z^{n_t + 259}]$	$z^{n_t+260}$
Testing	1	$[z^{1},..., z^{n_t-1}, z^{n_t}]$	$[z^{n_t+1},..., z^{449}, z^{450}]$

where *x* = the axial position, $x_A(t) = x_0-t\sqrt{gh_{up}}$, $x_B(t) = x_0 + t(\sqrt{gh_up}-3c_m)$ and $x_C = x_0+t\frac{2c_m^2(\sqrt{gh_up}-c_m)}{c_m^2-gh_{ds}}$, in which $c_m = \sqrt{gh_m}$ [28]. For each selected value in the generated sample set of the upstream water level, the analytical solution given above is evaluated over 1000 nodes ($n_s = 1000$) that contain the computational domain $x \, \in \, [0, 100]$ m for all 450 time-steps ($T = 450$) of the temporal domain $t \, \in \, [0, 3.6] s$. Four-hundred solution vectors, at random time steps, train the autoencoder network, and the remaining 50 vectors are used for the validation. The forecasting models are trained by the first 250 compressed samples and validated using subsequent 10 samples. During testing, the first $n_t$ latent vectors are utilized to predict vectors at subsequent time steps via auto-regression (Table 6).

#### Autoencoder for spatial compression

A similar methodology to the Burgers’ test case is adopted for the training of autoencoder models, AE and CAE, and forecasting models, LSTM, TCN, and CNN, for Stoker’s problem. The heat map plots obtained by stacking the predicted solution vectors along the x-axis (spatial nodes-$n_s$ along y, time-steps-$n_t$ along x), are illustrated for both autoencoder models in Appendix: Table 15.

Both AE and CAE effectively transform the solution vectors to a reduced latent space, since they produce fine reconstruction for vectors within as well as outside of the training domain of the autoencoder model. But again, only the CAE-CNN model learns the latent dynamics accurately enough to predict solution vectors outside the training domain (extrapolation).

The hyperparameters for the AE and CAE architectures are listed in Table 7.

**Table 7:** Stoker’s problem: hyperparameters for the AE and CAE networks

**Hyperparameters**	**MLP AE**	**Convolutional CAE**
Encoder layers	[500, 250]	[8, 32, 32]
Latent dimension (*m*)	25, 50, 125	25,50,125
Activation	relu, swish	relu, swish
Loss function	MSE	MSE
Learning rate	$10^{-3},3 \times 10^{-4}$	$10^{-3},3 \times 10^{-4}$
Model parameters ($m = 125$)	1,265,025	13,634

In the subsequent sections, the results of the forecasted model are produced using compression via the CAE with 3 layers having 8, 32 and 32 channels respectively in the encoder and a latent dimension of 125. The kernel size of the 1D convolution is 5, each layer has a padding of size 2. All the hidden layers use relu activation. A larger latent dimension is required for the Stokers’ case due to the discontinuities, as well as a larger domain size ($n_s = 1000$) as compared to the Burgers case. As the % compression increases, the loss of information increases, leading to inaccurate forecasts as well. As a result, a higher latent dimension is needed.

#### LSTM

The LSTM model receives input $Z \, \in \, {\mathbb {R}}^{m \times n_t}$ and predicts $z^{i+1}\, \in \, {\mathbb {R}}^{m}$ “Long short-term memory (LSTM)” section. The LSTM model has an architecture similar to that of the Burgers’ case, with multiple LSTM layers having a latent dimension of size 125 as their hidden dimension as well. The hyperparameters of the LSTM network for Stoker’s problem are summarized in Table 8.

**Table 8:** Stoker’s problem: hyperparameters for the LSTM network

**Hyperparameters**	**Values**
Sequence length ($n_t$)	5, 10, 20
LSTM layers	1, 2, 3
hidden/latent dimension (*m*)	25, 50, 125
Activation	tanh
Loss function	MSE
Learning rate	$5 \times 10^{-4}$
Max model parameters ($n_t = 20$)	126,000
Min model parameters ($n_t = 20$)	5200

**Fig. 15:**
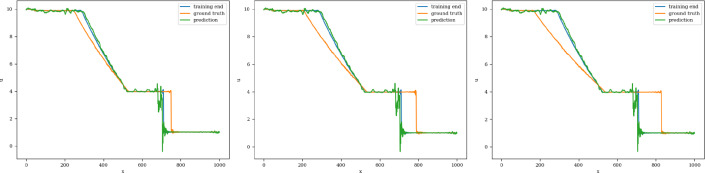
Stoker’s problem: extrapolative auto-regressive predictions of the LSTM model for time-steps = 310, 360, and 410; the end of training time-step = 260

**Fig. 16:**
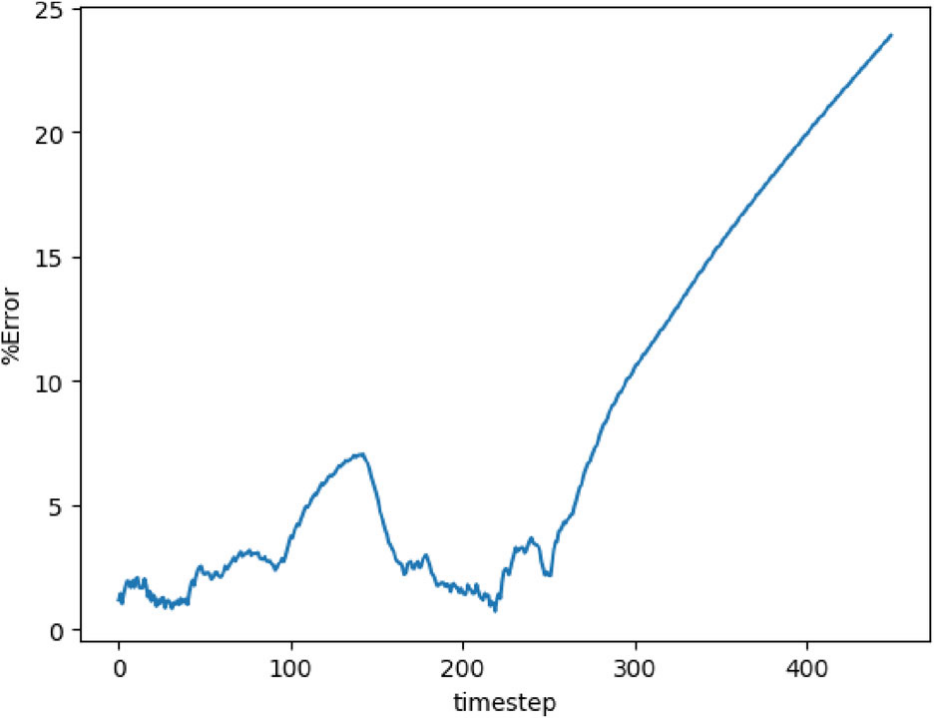
Stoker’s problem: $L_2$ relative error of the auto-regressive predictions with increasing time for the LSTM model

The models are trained in batches of size 15, and the loss values for both training and validation converge in 2400 epochs. The model with the least validation loss has a lookback window of size 10 and 2 LSTM layers with hidden dimensions of 125 each. The extrapolation (Fig. 15) and error plots (Fig. 16) obtained from these models show that the LSTM model accurately estimates the solution vectors for time-steps within the training domain ($i <= 250$), but fails outside the training domain, as the relative error reaches 25% for the 150th time-step post-training.

#### TCN model

The TCN model also receives a sequence $Z \, \in \, {\mathbb {R}}^{m \times n_t}$ and forecasts $z^{i+1}\, \in \, {\mathbb {R}}^{m}$, with the dilated convolutions operating on the temporal axis, and the latent dimension passed as a channel. The model contains three temporal blocks “Temporal Convolution Network (TCN)” section, with the same kernels and channels in each, and dilations that increase in size by a factor of two in subsequent blocks. The hyperparameters for the TCN network are listed in Table 9.

**Table 9:** Stoker’s problem: hyperparameters for the TCN network

**Hyperparameters**	**Values**
Sequence length ($n_t$)	5, 10, 20
TCN block channels	[100, 100, 100], [200, 200, 200]
Latent dimension (*m*)	25,50,125
Kernel size (k)	3, 5, 7, 9
Activation	tanh
Loss function	MSE
Learning rate	$3 \times 10^{-4}$
Max model parameters ($n_t = 20$, $m = 125$)	727,725
Min model parameters ($n_t = 20$, $m = 125$)	213,925

**Fig. 17:**
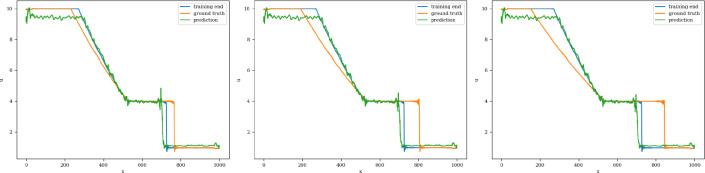
Stoker’s problem: Extrapolative auto-regressive predictions using the TCN model for time-steps = 320, 370, and 420. The training end is at time-step = 270)

**Fig. 18:**
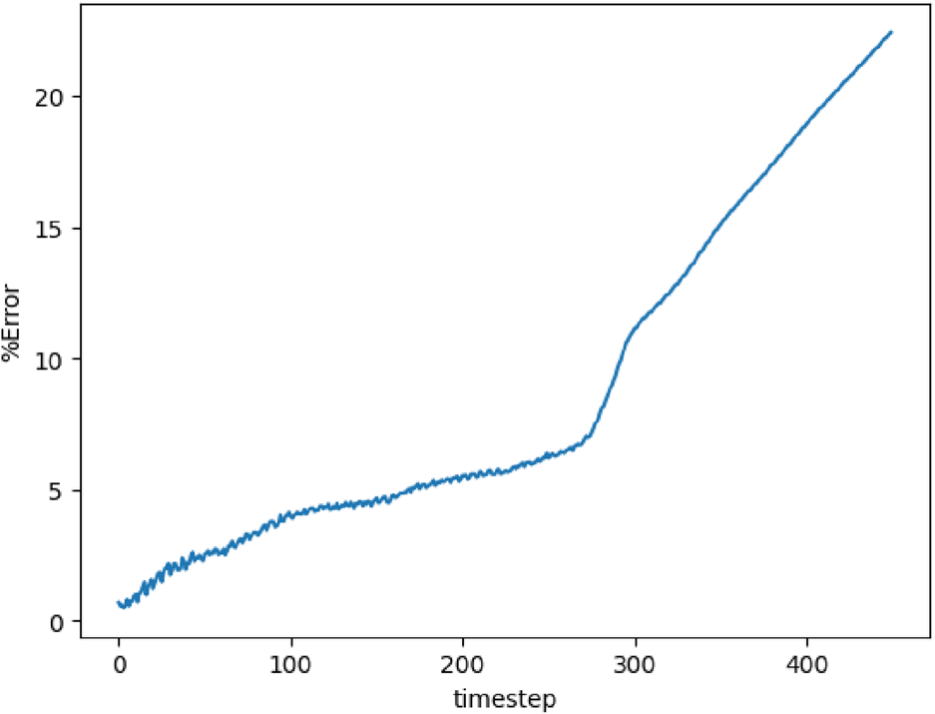
Stoker’s problem: $L_2$ relative error of the auto-regressive predictions with increasing time for the TCN model

Model training and validation loss reach convergence by 1000 epochs when training and validation are performed in batches of size 15. The best model (with the least validation loss) accommodates a sequence of vectors with a lookback window of size 20, and 125 latent modes. Each temporal block contains kernels of size 3 and has 200 channels. The extrapolation (Fig. 17) and error plots (Fig. 18) obtained from these models indicate that similar to the LSTM, the TCN also predicts the solution vectors with acceptable accuracy for time steps within the training domain ($i <= 250$), but the solution stops propagating further in the extrapolative domain, and so the error increases to 23%.

#### CNN model

The proposed CNN model takes the transposed vector sequence $Z^T \, \in \, {\mathbb {R}}^{n_t \times m}$ to produce $z^{i+1}\, \in \, {\mathbb {R}}^{m}$, with 1D convolutions operating on the spatial axis or latent dimension, and the $n_t$ latent vectors present on separate channels. The CNN model consists of three residual blocks “A proposed Convolution Neural Network (CNN) for time forecasting” section, each with the same kernel size and number of channels. The hyperparameters for the CNN model are summarized in Table 10:

**Table 10:** Stoker’s problem: hyperparameters for the CNN model

**Hyperparameters**	**Values**
Sequence length ($n_t$)	5, 10, 20
CNN block channels	[50, 50, 50], [100, 100, 100], [200, 200, 200]
Latent dimension (*m*)	25, 50, 125
Kernel size (k)	3, 5, 7, 9
Activation	tanh
Loss function	MSE
Learning rate	$3 \times 10^{-4}$
Max model parameters ($n_t = 20$, $m = 125$)	618,804
Min model parameters ($n_t = 20$, $m = 125$)	42,204

**Fig. 19:**
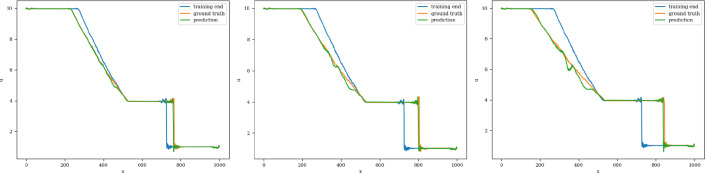
Stoker’s problem: extrapolative auto-regressive predictions using the CNN model for time-steps = 320, 370, and 420; the end of training is at time-step = 270

**Fig. 20:**
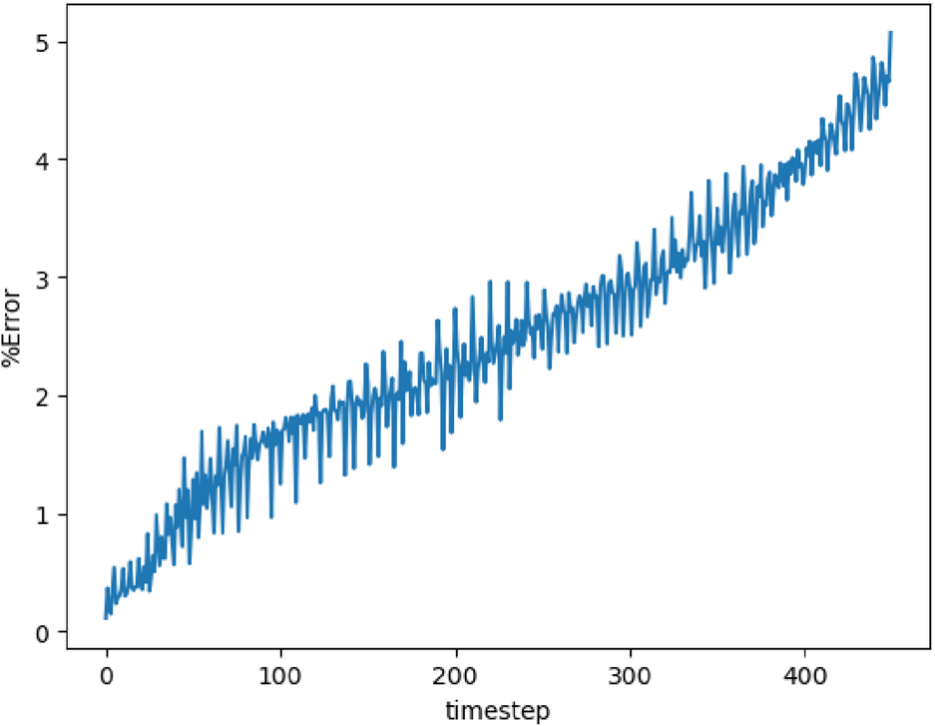
Stoker’s problem, $L_2$ relative error of the auto-regressive predictions with increasing time for the CNN model

The training and validation loss converges at around 1000 epochs when batches of size 16 are used. The model with the highest accuracy (least validation loss) has a lookback window of 20 steps, with 125 nodes in latent vectors at every step. Each of the three blocks possesses a kernel of size 3 and 100 channels in the 1D convolution layers. The extrapolation (Fig. 19) and error plots (Fig. 20) indicate that the CNN model is capable of modeling the latent dynamics since accurate forecasts are produced for time steps beyond the training domain. The error values increase over time, reaching 5%, which is remarkably lower than earlier models.

### Application to a hypothetical dam-break in a river

**Fig. 21:**
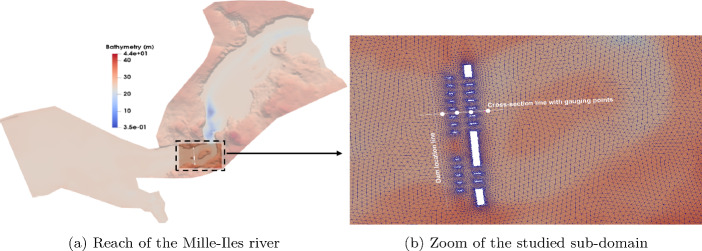
Sketch of the reach of the Mille-Iles River with a close-up view of the studied zone. The cross-section line and the gauging points represent locations where results are represented as a function of the longitudinal direction and time, respectively

To assess its effectiveness in conducting a forecasting analysis in a river with complex bathymetry, the most accurate (i.e., least relative error on extrapolation) forecasting model, i.e. the CNN future step predictor with a CAE autoencoder approach is implemented in this third test case. This test focuses on a section of the Milles-Iles River in the province of Québec, Canada, which includes a dam depicted in Fig. 21. The Communeauté Métropolitaine de Montréal (CMM) has provided the data on bathymetry and roughness coefficient based on measurements and observations. The study area consists of an unstructured triangular mesh comprising 16,763 elements and 10,200 nodes, where accurate solutions of the quantities of interest are obtained using an in-house multi-GPU finite volume solver specifically designed for shallow water equations [35]. For a comprehensive understanding of the physical domain of this test case, a detailed description was provided in [36].

An imaginary breach scenario was initiated in a hypothetical dam, as indicated by the line in Fig. 21b, where both sides have unequal water levels. The downstream section of the dam is assumed to be dry, while the free surface of the upstream section is treated as a random input parameter. The values of this parameter are uniformly generated within a plausible range of variability, denoted as $\eta _{up} \ \in \ {U (29, 32)}$ m. To create a snapshot matrix, the numerical solver is executed for each randomly selected value of the upstream free surface from the generated sample set. This process is carried out for 100 simulation time steps ($T = 100$) spanning the temporal domain ($t \in [0, 50] s$). For each combination of parameter and time, a high-fidelity solution is stored in a vector of dimension 10,200, representing the free surface values at each node in the computational domain.

The dataset for the 2D CAE-CNN architecture is generated after interpolating unstructured data vectors onto a background structured mesh, by using SciPy’s Griddata function. This interpolation technique is particularly effective since the data on nodes are irregularly spaced and do not form a regular grid suitable for CNN. The method parameter in the Griddata function specifies the interpolation method, which was set as ’linear’ in our case. The output of the Griddata function is an array of 2D solution matrices of dimension ${n_s \times n_s}$ containing the interpolated values from the unstructured mesh to the regular structured mesh.

Thus 100 solution matrix $[v^{1}, v^{2},..., v^{T}]$ of dimension ${n_s \times n_s} = 256 \times 256$ were generated via interpolation. These matrices are further compressed by the trained 2D CAE network to produce latent matrices $[z^{1}, z^{2},..., z^{T}]$ of dimension $m \times m = 32 \times 32$. Eighty solution matrices, at random time steps, train the 2D CAE network, and the remaining 20 vectors are used for the validation. The 2D CNN model is trained by the first 80 compressed samples and validated using the remaining 20. During testing, the first $n_t$ latent matrices are utilized to predict vectors at subsequent time steps via auto-regression (Table 11).

**Table 11:** 2D River problem: training, validation and testing dataset

**Dataset**	**Samples**	**Input**	**Output**
Training	1	$[z^{1},..., z^{n_t-1}, z^{n_t}]$	$z^{n_t+1}$
	2	$[z^{2},..., z^{n_t}, z^{n_t+1}]$	$z^{n_t+2}$
	...	...	...
	50	$[z^{50},..., z^{n_t+48}, z^{n_t+49}]$	$z^{n_t+50}$ (training end)
Validation	51	$[z^{51},..., z^{n_t+49}, z^{n_t+50}]$	$z^{n_t+51}$
	...	...	...
	60	$[z^{60},..., z^{n_t+58}, z^{n_t+59}]$	$z^{n_t+60}$
Testing	1	$[z^{1},..., z^{n_t-1}, z^{n_t}]$	$[z^{n_t+1},..., z^{99}, z^{100}]$

#### 2D Convolutional autoencoder for spatial compression

A similar methodology to previous test cases is adopted for training the 2D CAE model. The model also has a similar architecture, with the encoder consisting of three 2D convolution layers, each followed by activation and 2D max pooling layers. Another 2D convolution layer is used at the end to obtain a 2D latent vector consisting of a single channel. The latent vector obtained is not further compressed or flattened to a single dimension and the model is also devoid of any dense layer. Therefore during compression, the local spatial information is preserved to facilitate the modeling of latent dynamics by the CNN model since it convolves on the spatial axis of the matrix and utilizes information from the neighboring nodes to predict the future timestep value at a specified node. The decoder has a mirror architecture to the encoder.

The 2D CAE effectively transforms the 2D solution matrix to a reduced 2D latent space of dimension 32 $\times$ 32, since they produce fine reconstruction for solution matrices within as well as outside of the training domain of the model. The hyperparameters for the employed 2D CAE architecture are listed in Table 12.

**Table 12:** 2D River problem: hyperparameters for the 2D CAE network

**Hyperparameters**	**2D convolutional CAE**
Encoder layers channels	[16, 32, 16]
padding mode	Replicate
Latent dimension (*m*)	32x32
Activation	relu
Loss function	MSE
Learning rate	$3 \times 10^{-4}$
Model parameters ($m = 32 \times 32$)	19,138

#### 2D CNN model

The 2D CNN model consists of three residual blocks “A proposed Convolution Neural Network (CNN) for time forecasting” section, each having two 2D convolution layers, with the same kernel size and number of channels. It takes the transposed vector sequence $Z^T \, \in \, {\mathbb {R}}^{n_t \times m}$ to produce $z^{i+1}\, \in \, {\mathbb {R}}^{m}$, with 2D convolutions operating over 2D spatial domain, and the $n_t$ latent matrices present on separate channels. The hyperparameters for the CNN model are summarized in Table 13:

**Table 13:** 2D River problem: hyperparameters for the CNN model

**Hyperparameters**	**Values**
Sequence length ($n_t$)	20, 30
CNN block channels	[200, 200, 200], [250, 250, 250], [300, 300, 300]
Latent dimension (*m*)	32 $\times$ 32
Kernel size (k)	3
Activation	tanh
Loss function	MSE
Learning rate	$3 \times 10^{-4}$
Max model parameters ($n_t = 30$)	4,144,204
Min model parameters ($n_t = 30$)	1,862,804

**Fig. 22:**
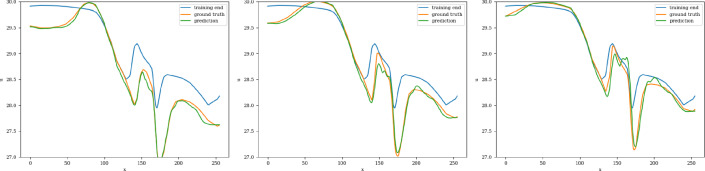
2D River problem: Interpolative auto-regressive predictions using the CNN model for 20th, 30th, and 40th timestep after the first initial 30 time-steps used in the lookback window (i.e. timestep = 50, 60, and 70 respectively); the end of training is at time-step = 80

**Fig. 23:**
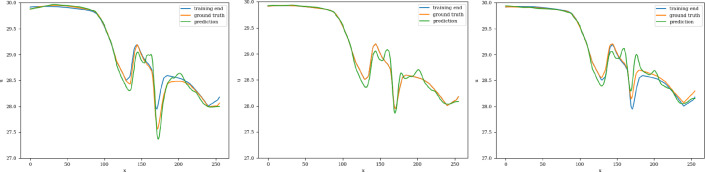
2D River problem: Extrapolative auto-regressive predictions using the CNN model for time-steps = 81, 90, and 99; the end of training is at time-step = 80

**Fig. 24:**
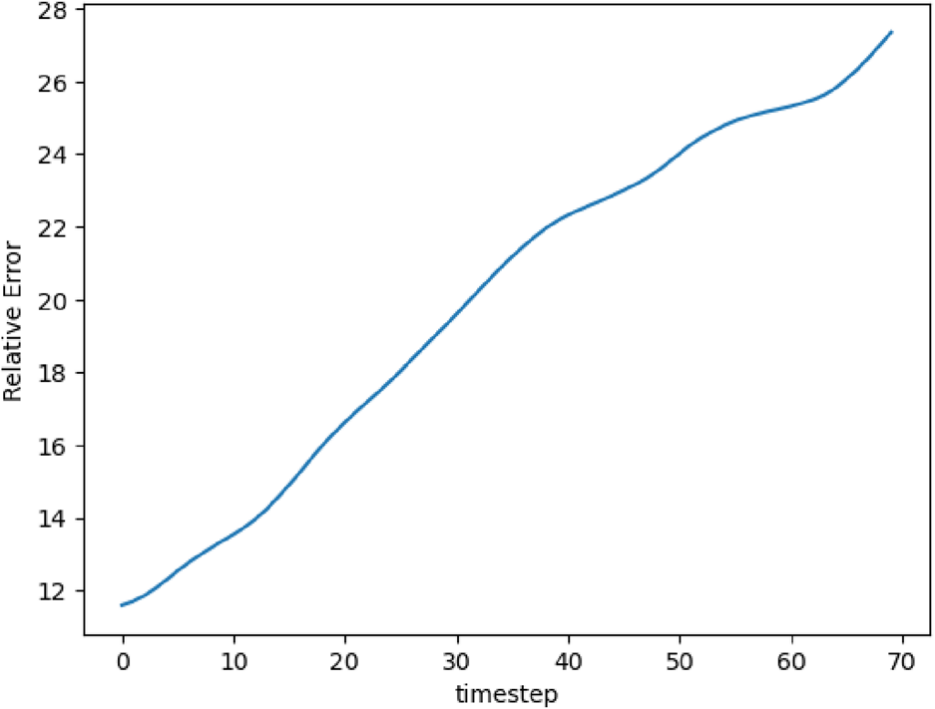
2D River problem, $L_2$ relative error of the auto-regressive predictions with increasing time for the CNN model

The training and validation loss converges at around 2000 epochs when batches of size 16 are used. The model with the highest accuracy (least validation loss) has a lookback window of 30 steps, with the latent matrix having a dimension of 32 $\times$ 32. Each of the three blocks possesses a kernel of size 3 and 200 channels in the 2D convolution layers. The interpolation curves (Fig. 22) produced by the 2D CNN model are highly accurate and follow the ground truth as time progresses. The extrapolation prediction (Fig. 23) further stipulates the capability of the proposed architecture in modeling the dynamics of the 2D dam-break scenario, since reasonable forecasts are produced for time steps beyond the training domain as well. The error values (Fig. 24) gradually increase over time (as expected), reaching 27% at the 100th time-step. These errors can be explained (at least partially) by the fact that when the water propagates over the dry land, no physical information, such as the evolution of the bathymetry, is provided to the model on the downstream side. Therefore, even if the predictor CNN has more potential than the well-known LSTM or TCN, the forecasting problem may be ill-posed and manifests by the shown instabilities.

## Conclusion

This study proposes a Convolutional Autoencoder (CAE) model for compression and a CNN future-step predictor for forecasting vector solutions for subsequent time steps to input vector sequences. The approximation accuracy and time extrapolation capabilities of the model are evaluated using three advection-dominated flow problems, Burgers’ and Stoker’s problems, as well as the hypothetical dam break problem in a real river having a complex 2D bathymetry. All the problems are characterized by sharp gradients. The models built especially for time-series forecasts, LSTM and TCN models with propagation over time, produce acceptable results within the training domain, but the solution stops changing during extrapolation. However, when the dilated convolutions propagate on the spatial axis, the models produce good predictions for extrapolation as well.

The proposed CNN model for forecasting has an architecture (residual blocks with 1D convolutions propagating along space) similar to that of the TCN model but without causal padding or dilation. These have been eliminated, as the increasing receptive field has no significance during convolution in space, and causal padding degrades the results by causing the information extracted from neighboring cells to be shifted or scraped. The CNN model is capable of producing highly accurate predictions for both the 1D test cases, with less than 5% relative $L_2$ error in the extrapolation domain ($\approx$ 60% of the training time domain). The results produced by the 2D CAE-CNN model for the dam break problem are also acceptable with a relative $L_2$ error under 28% in the extrapolation domain ($\approx$ 25% of the training time domain). However, the CNN models (both 1D and 2D) only produce accurate forecasts if they receive compressed latent vectors from the CAE models (1D and 2D respectively) since they preserve the local spatial information better than the MLP encoder during compression. In addition, since the CNN model uses convolutions (like TCN [34]), the training and evaluation can be done in parallel for long input sequences, in contrast to the LSTM. Thus, a fast, accurate, and robust framework is provided for order reduction. The model architecture is flexible and can be extended for three-dimensional spaces as well, by increasing the dimension of the convolutional filters. Future work will focus on improving the model by using physical mechanisms to inform the model during the forecasting phase and considering probabilistic settings to treat the uncertainties.

## Figures and Tables

**Table 14:**
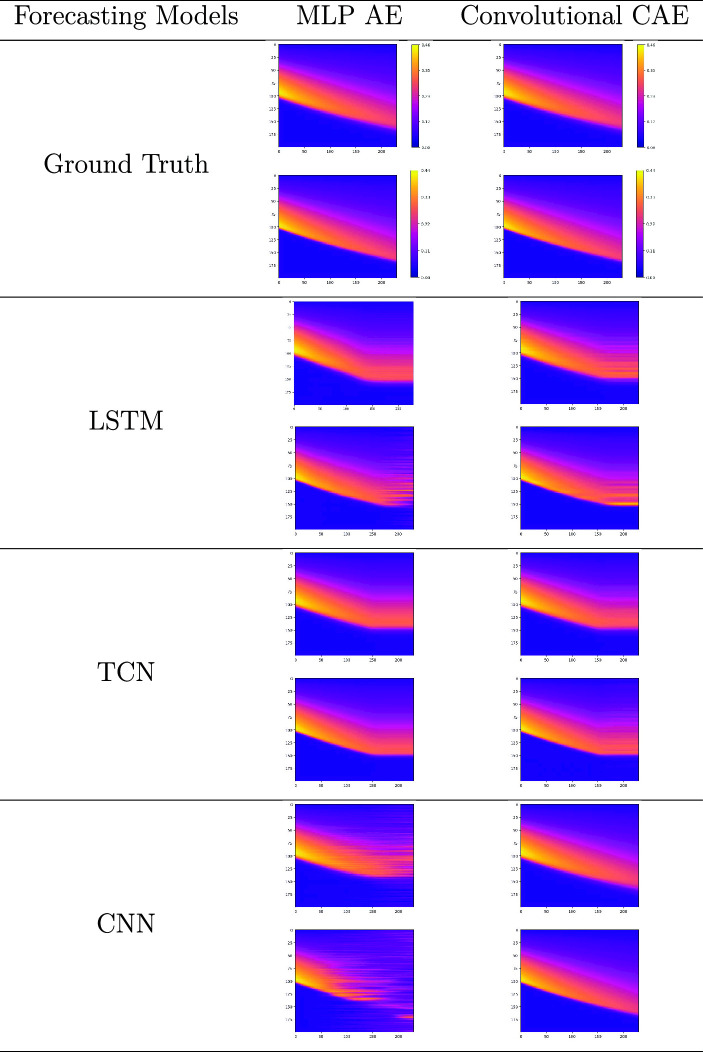
Burgers’ problem: auto-regressive forecasts for Re= 300 (top) and Re= 600 (bottom) from the forecasting models when latent vectors are obtained by AE and CAE

**Table 15:**
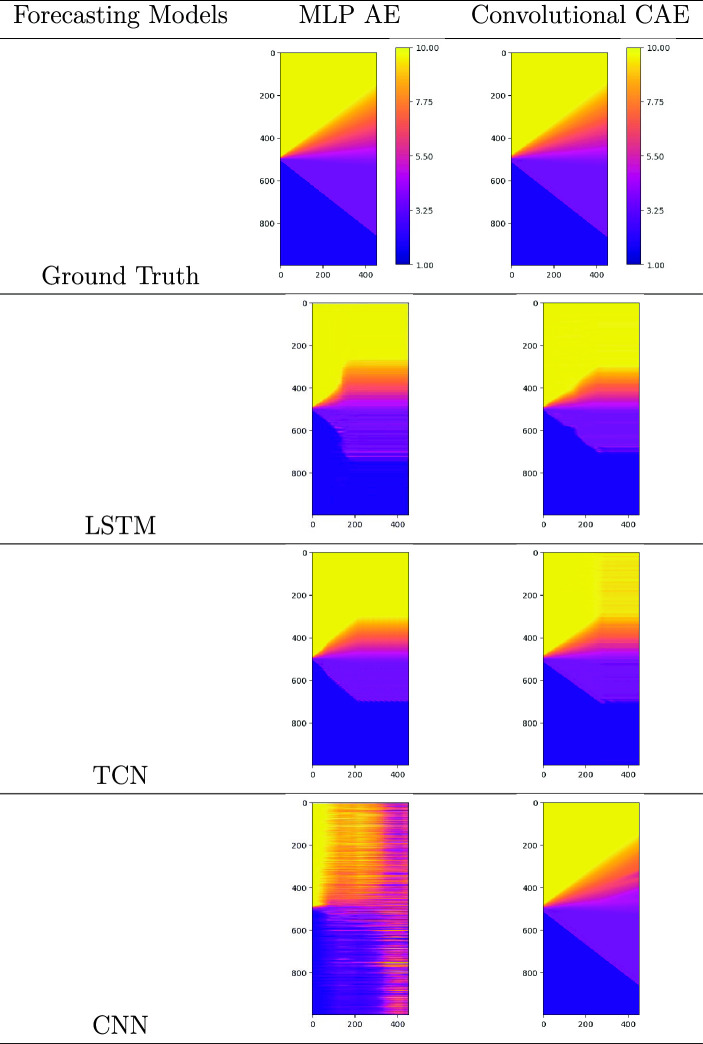
Stokers’ problem: auto-regressive forecasts from the forecasting models when latent vectors are obtained by AE and CAE

## Data Availability

All the source codes developed for this study are available at https://github.com/Yash-11/lstm_ROM.

## Appendix: Heat map plots for Burgers’ and Stoker’s problems

See Tables 14, 15.
